# Prognostic potential of whole exome sequencing in the clinical management of metachronous colorectal cancer liver metastases

**DOI:** 10.1186/s12935-023-03135-x

**Published:** 2023-11-26

**Authors:** Lucie Heczko, Viktor Hlaváč, Petr Holý, Pavel Dvořák, Václav Liška, Ondřej Vyčítal, Ondřej Fiala, Pavel Souček

**Affiliations:** 1 Laboratory of Pharmacogenomics, Biomedical Center, Faculty of Medicine in Pilsen, Charles University, Pilsen, 306 05 Czech Republic; 2 Toxicogenomics Unit, National Institute of Public Health, Prague, Czech Republic; 3 Department of Biology, Faculty of Medicine in Pilsen, Charles University, Pilsen, Czech Republic; 4 Department of Surgery, Faculty of Medicine and University Hospital in Pilsen, Charles University, Pilsen, Czech Republic; 5 Department of Oncology and Radiotherapeutics, Faculty of Medicine and University Hospital in Pilsen, Charles University, Pilsen, Czech Republic

**Keywords:** Exome, Colorectal cancer, Liver Metastasis, Metachronous, Therapy, Prognosis

## Abstract

**Background:**

Colorectal cancer is a highly prevalent and deadly. The most common metastatic site is the liver. We performed a whole exome sequencing analysis of a series of metachronous colorectal cancer liver metastases (mCLM) and matched non-malignant liver tissues to investigate the genomic profile of mCLM and explore associations with the patients’ prognosis and therapeutic modalities.

**Methods:**

DNA samples from mCLM and non-malignant liver tissue pairs (*n* = 41) were sequenced using whole exome target enrichment and their germline and somatic genetic variability, copy number variations, and mutational signatures were assessed for associations with relapse-free (RFS) and overall survival (OS).

**Results:**

Our genetic analysis could stratify all patients into existing targeted therapeutic regimens. The most commonly mutated genes in mCLM were *TP53*, *APC*, and *KRAS* together with *PIK3CA* and several passenger genes like *ABCA13, FAT4, PCLO*, and *UNC80.* Patients with somatic alterations in genes from homologous recombination repair, Notch, and Hedgehog pathways had significantly prolonged RFS, while those with altered MYC pathway genes had poor RFS. Additionally, alterations in the JAK-STAT pathway were prognostic of longer OS. Patients bearing somatic variants in *VIPR2* had significantly shorter OS and those with alterations in *MUC16* prolonged OS. Carriage of the *KRAS*-12D variant was associated with shortened survival in our and external datasets. On the other hand, tumor mutation burden, mismatch repair deficiency, microsatellite instability, mutational signatures, or copy number variation in mCLM had no prognostic value.

**Conclusions:**

The results encourage further molecular profiling for personalized treatment of colorectal cancer liver metastases discerning metachronous from synchronous scenarios.

**Supplementary Information:**

The online version contains supplementary material available at 10.1186/s12935-023-03135-x.

## Background

Colorectal cancer (CRC) is the second leading cause of cancer-related deaths and the third most common cancer in the world [1]. While the 5-year survival rate in the USA for stage I is 92% and for stage II to III 90 − 72%, the survival rate for stage IIIC it is 53% and for stage IV metastatic CRC it is only 12% [2]. Surgery remains the first choice for localized stage CRC. However, about 25% of patients who underwent radical surgery for CRC develop metastases [3], and these patients have a markedly worse prognosis since systemic therapies are less effective owing to the rapid evolution of cancer resistance [2].

Metachronous colorectal liver metastases (mCLM), i.e., metastases diagnosed more than six months after the primary cancer surgery vary in prognosis and molecular background from synchronous metastases (i.e. those ones that are diagnosed at the time of diagnosis or during the therapy) [4, 5]. A recent study reported that patients with synchronous (*n* = 215) CRC liver metastases had significantly shorter median overall survival compared to those with mCLM (18.5 versus 62.8 months; p value < 0.001) and lower CEA oncomarkers levels [4, 5].

MCLM patients can be treated with surgery including liver resection or locally ablative methods such as RFA or MWA. Unresectable metastases can be converted to resectable by neoadjuvant systemic treatment or remain, leading to continuation of palliative systemic therapy as the final choice [6]. The success of metastatic CRC treatment depends on the molecular subtype, patient comorbidities, and performance status. The mutations in *RAS/BRAF* and MSI or MRR-D statuses are currently the most important molecular predictive biomarkers for choosing the type of systemic therapy, influencing the overall survival (OS) [3]. The median OS of the metastatic CRC patients treated with systemic therapies ranges between 19 and 30 months. The extent to which the full spectrum of genetic variability accounts for the differences in prognosis between individual patients is currently unknown. About half of patients with wild-type *RAS/BRAF* do not respond well to the anti-EGFR therapy [3], and for the 40% of patients with mutations in *RAS/BRAF*, the landscape of targeted therapy is currently evolving. Knowledge of the full genomic background might therefore help understand the biological processes behind metastatic formation and provide biomarkers of metastatic CRC patients’ prognosis and therapy response.

Initial studies identified frequent mutations and recurrent copy number variants (CNVs) by exome or genome sequencing of CRC patients, hinting at processes involved in distant metastasis formation [7]. Recently, a single-cell exome sequencing study of primary tumors, proximal normal tissue, and colorectal liver metastases (CLM) in several patients revealed evolutionary subclones [8]. Other studies analyzed the genetic landscape of CLM by whole exome sequencing of DNA extracted from bulk tumor tissues or sections. One study identified *TTN*, *OBSCN*, and *HYDIN* as the most mutated in CLM of four patients, and GO analysis showed that affected pathways included cell, cell part, and cellular process, while KEGG pathways included gastric acid secretion, bile secretion, and melanogenesis [9]. Another study identified the common clonal origin of two lesions, with recurrently mutated *KRAS, SYNE1, CACNA1H, PCLO, FBXL2*, and *DNAH11*, and showed that the 8q amplification CNV event was specific for metastasis [10]. Feng et al. [11] found that *TP53*, *APC*, and *KRAS* were the top mutated genes in eight patients of Chinese origin. Moreover, genes could be classified into five major categories with binding and catalytic activity having the most “molecular function” hits. In addition, affected pathways included Wnt, angiogenesis, p53, Alzheimer’s disease-presenilin, Notch, and cadherin signaling. Nevertheless, a study comparing the complex genomic profile of mCLM, separately from the synchronous metastasis scenario, and evaluating the importance of variability in surrounding non-malignant liver tissues in a larger cohort of patients with complete clinical follow-up, is still missing.

Although patient survival can be improved with targeted treatment, there is no reliable biomarker for the risk of patient progression after CRC liver metastasis surgery and therapy with curative intent, especially one that would recognize the two distinct metastatic scenarios (synchronous and metachronous). We aimed to provide the first dataset to investigate the complex molecular profile, including single nucleotide variants (SNVs), small insertions-deletions (indels), CNVs, and mutational signatures of mCLM and paired non-malignant liver tissue samples connected with relevant clinical information, including survival of patients. Here, we present the exome profiles with prognostic meaning based on the relapse-free survival (RFS) and OS of patients and subsequent therapeutic considerations for therapy of recurrences after radical mCLM surgery.

## Methods

### Patients

Paired samples of surgically resected mCLM and non-malignant liver tissues were collected from 41 patients who were previously treated for their primary CRC tumors. Metastasis diagnosed at least 6 months or later after the surgery of the primary CRC was considered metachronous [12]. All patients were operated on at the Department of Surgery of the University Hospital in Pilsen between 2012 and 2017. The clinical data including age, gender, date of primary and mCLM diagnosis, data concerning mCLM surgery and oncological treatment, date of recurrence or progression after mCLM surgery, and date of last control or death were obtained from medical records. The patients’ demographic and clinical characteristics are summarized in **Supplementary Table ****S1**. The OS was defined as the time elapsed between mCLM resection and death from any cause or patient censoring. The RFS was defined as the time elapsed between the mCLM resection and subsequent disease relapse; death or last control in remission were censored events. The study protocol was approved by the Ethical Commission of the Faculty of Medicine and University Hospital in Pilsen (approval no. NT12025-4 of 16 September 2010). All patients provided their informed consent with the study participation.

### DNA isolation and quantification

DNA from fresh-frozen tissue samples of mCLM and non-malignant liver tissue was isolated with the DNeasy Blood and Tissue Kit (Qiagen, Hilden, Germany) according to the manufacturer´s instructions. DNA was eluted into 200 µL of AE buffer, divided into triplicates and stored at − 20 ^o^C until further use.

The isolated DNA was quantified using Qubit 3.0 Fluorometer and dsDNA High Sensitivity Assay Kit (both ThermoFisher Scientific, Waltham, MA, USA). The purity of DNA was assessed as the ratios of A260/280 and A260/230 using NanoDrop 1000 (ThermoFisher Scientific).

### Library preparation and whole exome sequencing

The libraries for sequencing were prepared using SureSelectXT HS2 System (Agilent, Santa Clara, CA, USA) according to the manufacturer´s instructions. Briefly, 100 ng of tumor DNA was enzymatically digested, ends of sequences were repaired, adaptors were ligated, and the libraries were amplified using four ligand-mediated PCR cycles. The quality of prepared libraries was controlled using TapeStation 2200 (Agilent) and libraries were quantified using Qubit 3.0 Fluorometer and dsDNA High Sensitivity Assay Kit (ThermoFisher Scientific).

Samples were multiplexed in pools each containing seven libraries derived from either metastasis or liver DNA and hybridized using SureSelect Human All Exon V8 probes (SureSelectXT HS2 System, Agilent). Captured sequences were amplified by 10 post-LM-PCR cycles and their quality was assessed using TapeStation 2200 (Agilent). Libraries were quantified by Qubit and pooled in a non-equimolar fashion (tumors/normal liver ratio 5:1). Final pool of samples was sequenced on the NovaSeq 6000 platform (Illumina, San Diego, CA, USA) using 150 bp pair-end sequencing on one lane of the S4 flow cell.

### Bioinformatic analysis

The bioinformatics pipeline used for raw data processing has been described elsewhere in detail [13]. Below, we describe the procedure only briefly with relevant references.

### Raw data processing and variant detection

Reads were aligned to the hg38 human reference genome sequence using Burrows-Wheeler Aligner v0.7.17-r1188 (BWA, Cambridge, UK) with the BWA-maximal exact matches (MEM) algorithm [14]. Base recalibration was done using the Genome Analysis Toolkit v.4.2.6.1 (GATK) (Broad Institute, Cambridge, UK) according to GATK Best Practices [15]. Identification of somatic variants and short indels was performed in paired tumor-normal samples using Mutect2 (GATK). Detected variants were filtered using FilteredMutectCalls (GATK) and only variants passing all filters (i.e., somatic variants with filter status PASS) were considered. Annotation was performed in Variant Effect Predictor (VEP) v.108, which assigned one of the following values to each variant: LOW, MODIFIER, MODERATE, or HIGH functional effect. Germline variants were called using Haplotype Caller (GATK). Variants were considered rare and deleterious if they had allele frequency in gnomAD < 0.05 and had either predicted “HIGH” impact by VEP (protein loss-of-function, e.g., stop-gain, stop-loss, frameshift insertion or deletion, etc.), CADD score > 25 [16] or were listed as pathogenic in ClinVar [17].

For mutational signature analysis, we utilized the R Bioconductor package sigminer v2.2.0 [18] to assess the contribution of each of the 79 reference SBS signatures in the COSMIC database (version 3.3, June 2022) in each sample using the *sig_fit* function with the detection cut off set to 0.05 of relative exposure. From the first refitting, the top 10 signatures by overall contribution were selected, from which one signature was eliminated due to being a suspected sequencing artefact by COSMIC (SBS54), and a second refitting was performed with only the remaining 9 signatures and the decline in the quality of the fit was assessed by calculation of cosine similarity [18]. Tumor mutation burden (TMB) was defined as the number of non-silent mutations (“HIGH” or “MODERATE” functional effect) per Mb [19] with 10% cut-off for TMB-high samples.

CNVs were detected with CNVkit v0.9.9 [20] and VarDict tool v1.8.3 [21]. Tumor purity was estimated using PureCN v.2.0.2 (R/Bioconductor). Significant calls were assessed based on the average read depth log2 ratio values and B-allele frequencies (BAF) of individual segments. Assuming theoretical clonal fraction (tumor purity) of 70%, a deletion should have log2 ratio < -0.278 and BAF between 0.325 and 0.675; a duplication should have log2 ratio > 0.233 and BAF between 0.442 and 0.558. All called segments that contained less than three bins or did not show a statistically significant difference of log2 ratios compared to reference values (*p* < 0.05 by the Student’s t-test) were excluded.

Microsatellite instability was detected using MSIsensor2 v0.1 (https://github.com/niu-lab/msisensor2) based on the published 20% threshold [22]. Homopolymer regions were identified by Vcfpolyx (part of Jvarkit, https://github.com/lindenb/jvarkit) and were defined as genomic regions with more than four repeat bases. MMR-D was calculated based on the cut-off set to 1.5 indels in homopolymer regions per Mb [19].

### Annotation and interpretation of detected variants

Detected somatic variants were annotated and converted into the mutation annotation format (MAF) using vcf2maf v.1.6.21. For comparisons of mutation rates between patient groups and for the creation of somatic variant plots, the maftools 2.12.0 R/Bioconductor package was used [23].

### External validation

For validation of findings in *KRAS*, we used a previously published cohort of metastatic CRC patients (“MSK cohort”, [24]) with panel sequencing. This dataset contains results of somatic profiling of 1134 CRC patients and enables discerning of profiles from primary tumors and metastases (*n* = 533), together with the location of metastatic spread. First, we filtered down the dataset to only those samples that were of metastatic tissue resected from liver as the only site of first metastasis (statuses Biopsy = liver or Resection = 1 (liver as the only site of first metastasis)). We limited patients to only those with disease stage I-III, since those would be expected to have metachronous metastases, as opposed to stage IV patients, which would have synchronous metastases. This resulted in a cohort of 97 samples with complete OS data. Gene names were converted into Human Genome Organisation Gene Nomenclature Committee (HGNC) using BioMart (https://www.ensembl.org/info/data/biomart/index.html).

### Statistical analyses

Differential analyses were performed in patient subgroups stratified by survival status. Analyses of differences in numbers of variants between groups of patients divided by the 6-month cut-off were performed using Fisher’s exact test. Differences in mutational signatures and CNVs between patients stratified by survival status were compared using Pearson’s chi-square test (for factorial comparisons) or the Mann-Whitney test (factorial vs. continuous data). Correlations of continuous data such as patient age, CNVs size or CNV counts were assessed using Spearman’s rho test.

Survival functions for groups of patients divided by genetic data were plotted using the Kaplan-Meier method and significance was calculated by the Log rank test. All continuous variables were divided by the median (for mutational signatures relative exposure < 0.05 was considered to be zero).

A two-sided p-value of < 0.05 was considered significant. All statistical analyses were performed in SPSS v16 program (SPSS Inc., Chicago, IL, USA) or R.

## Results

### Clinical characteristics of the patients

The summary of the main characteristics of all patients is in **Supplementary Table ****S1**. The median age at the time of mCLM diagnosis was 65 years (range 37–78) and the group contained more men (61%) than women (39%). This unequal sex distribution is in agreement with the reported higher CRC incidence in men compared to women [1]. After the curative mCLM surgery, the median of RFS was 11 (range 1–103) months. Seven patients experienced no progression after mCLM treatment and except one had long OS > 3 years. The reported median OS of patients with CRC liver metastases after curative surgery is 24 to 37 months in the last ten years [25]. In agreement with previous studies, the median OS of our dataset was 40 (3–103) months. After mCLM resection, patients were treated predominantly with regimens FOLFOX, CAPOX, FOLFIRI, OR CAPIRI alone or with targeted therapy (cetuximab, panitumumab, or bevacizumab based on *RAS/BRAF* mutation screening results). Patients survival between primary tumor and mCLM resection was not affected by pTNM, stage, or grade of primary tumor or by administration of adjuvant treatment (none vs. administered). Survival between mCLM resection and second relapse or death was unaffected by the above-mentioned factors, the mCLM resection radicality, or administered chemotherapy after mCLM resection (*p* > 0.05). A trend towards a better outcome of the patients with single compared to those with two or more mCLM loci was observed (*p* = 0.08).

### General description of the whole exome profiling

The average coverage was 238x for mCLM and 68x for non-malignant liver. On average, 83% of bases in metastases (41% for non-malignant tissue) were covered at least 30x, and 94% (88%, respectively) at least 10x. The duplicate rate was 53% for metastases and 37% for non-malignant liver tissue.

### Somatic profile of mCLM

**Fig. 1:**
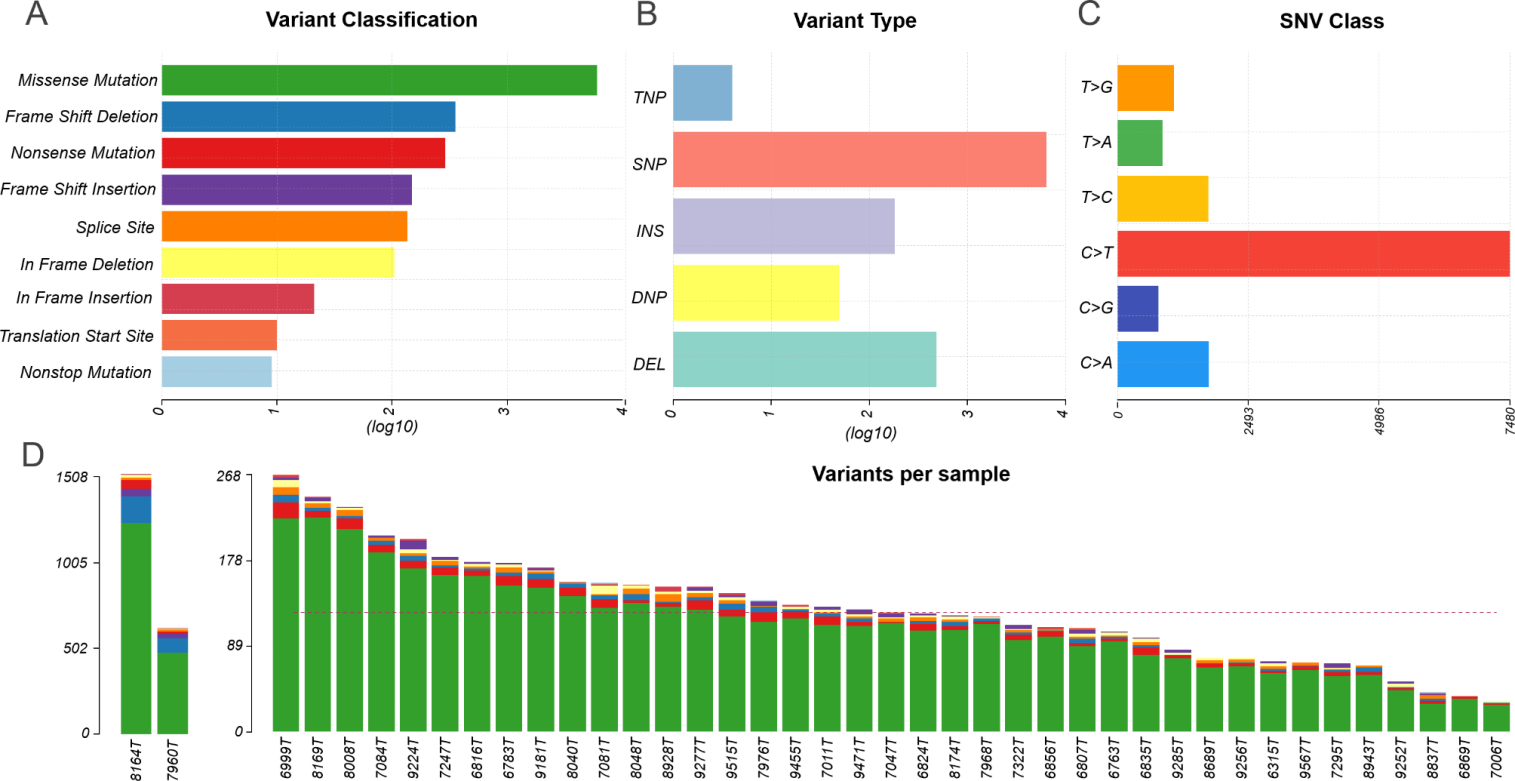
The summary of the distribution of the overall variants in mCLM. Only protein-changing variants were considered, 7 952 silent variants were excluded from the analysis. **(A)** The classification of variants according to their functional effect (missense mutation, frameshift deletion/insertion, nonsense mutation, splice site, inframe deletion/insertion, translation start site, or nonstop mutation). On the x-axis, the counts are in the log scale. The most prevalent variants were missense. **(B)** The types of variants (TNP stands for trinucleotide variant; SNP, single nucleotide polymorphism; INS, insertion; DNP, dinucleotide variant; DEL, deletion). On the x-axis, counts are in the log scale. The most common type of variant was the SNP. **(C)** The type of nucleotide substitution. The most frequent substitution was the C > T transition. **(D)** The counts and distribution of the variants for the indicated samples; the dashed line represents a median (124 variants per sample excluding silent variants). Two TMB-H patients are separated to deflate the y-axis

The total number of detected variants per mCLM sample was 1 528 ± 798 on average (ranging from 966 to 5 292, median 1 388). The amount of somatic variants fulfilling the filtering criteria (see Materials and Methods) per sample was 370 ± 464 (ranging from 82 to 2 932, median 281). From these, 7 952 silent variants were then excluded from downstream analyses. The distribution of pathogenic somatic variants in gene coding regions among 41 patients in our cohort is shown in Fig. 1. The most common class of somatic variants was the missense mutation (Fig. 1A), and the most common type was a single nucleotide variant (SNV, Fig. 1B). The most common nucleotide substitution was the C > T transition (Fig. 1C). Two patients had TMB-high status by definition and notably differed by the mutational load (total numbers of non-silent variants were 1 508 and 616, respectively) from the rest (Fig. 1D). The overall mutational summary for all samples is in **Supplementary Table ****S2**. From the list of 20 FLAGS (FrequentLy mutAted GeneS) genes that are known to be frequently mutated in cancer but are unlikely to be pathogenic [26], *TTN*, *AHNAK2*, *SYNE1*, *MUC16*, and *OBSCN* were found among genes altered at ≥ 20% in mCLM. Due to their FLAGS status, these genes will not be discussed further.

**Fig. 2:**
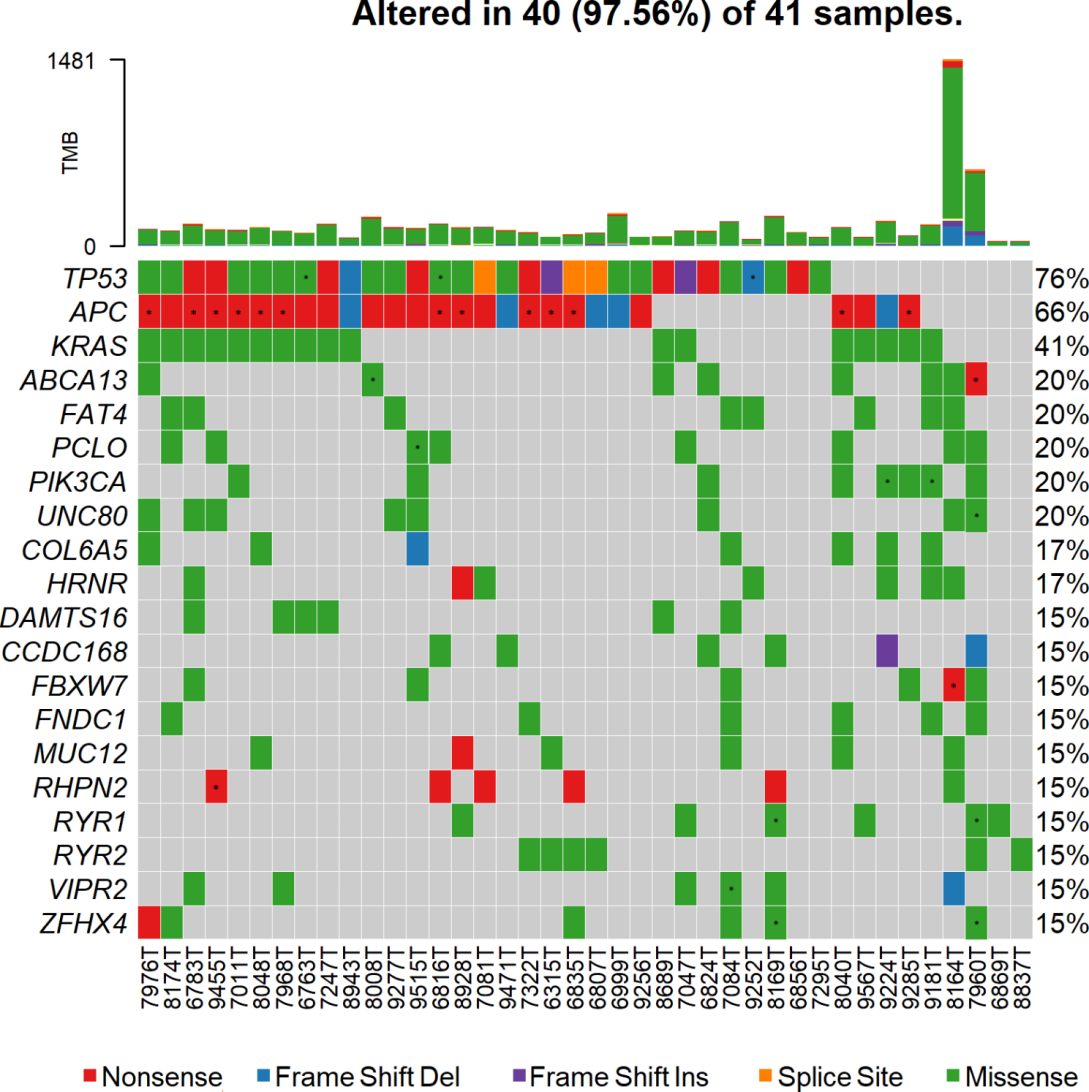
Oncoplot of most mutated 20 genes (Top 20)

From the rest of the genes, the most frequently mutated gene was *TP53*, which was detected in 76% of patients (31/41; 34 variants in total) (Fig. 2). Missense was the most common variant type in *TP53*, followed by nonsense, frameshift indels, and splice site variants (lollipop in **Supplementary Fig. ****S1****B**). The second most frequently mutated gene was *APC*, with variants detected in 66% of patients (27/41; 42 variants in total) in our cohort (Fig. 2). The most frequent *APC* alterations were nonsense variants followed by frameshift deletions, missense variants, and frameshift insertions (lollipop in **Supplementary Fig. ****S1****A**). In other genes, e.g., *KRAS* the missense variants prevailed (lollipop in **Supplementary Fig. ****S1****C**).

Analysis of mutation co-occurrence (**Supplementary Fig. ****S2**) revealed that *ABCA13* were altered together with *MYT1L* (*p* < 0.01), *FREM2*, *UNC80*, and *PIK3CA* (all *p* < 0.05). Variants in *UNC80* also co-occurred with alterations in *FBXW7* (*p* < 0.01) and *PCLO* (*p* < 0.05). Furthermore, the alterations in *FREM2* co-occurred with variants in *PIK3CA*, *COL6A5*, and *HRNR* (all *p* < 0.05) and those in *HRNR* with alterations in *EYS* and *FAT4* (both *p* < 0.05). Moreover, alterations in *TP53* and *FREM2* were mutually exclusive (*p* < 0.01). Interestingly, alterations in *TP53* and *PIK3CA* and in *KRAS* and *RYR2* were mutually exclusive too (*p* < 0.05).

We then performed an analysis of CNV in mCLM samples. On average, the tumors bore 55.9 ± 30.4 CNVs (ranging from 16 to 151, median 53). The average size of the deletions/amplifications was 11.7 ± 5.0 Mb. Most common CNVs were single-copy amplifications (23.3% of all CNVs), followed by single-copy deletions (21.8%). Less common were CNVs with > 3 copies (9.5%) and homozygous losses (1.2%) (**Supplementary Table S4**).

**Fig. 3:**
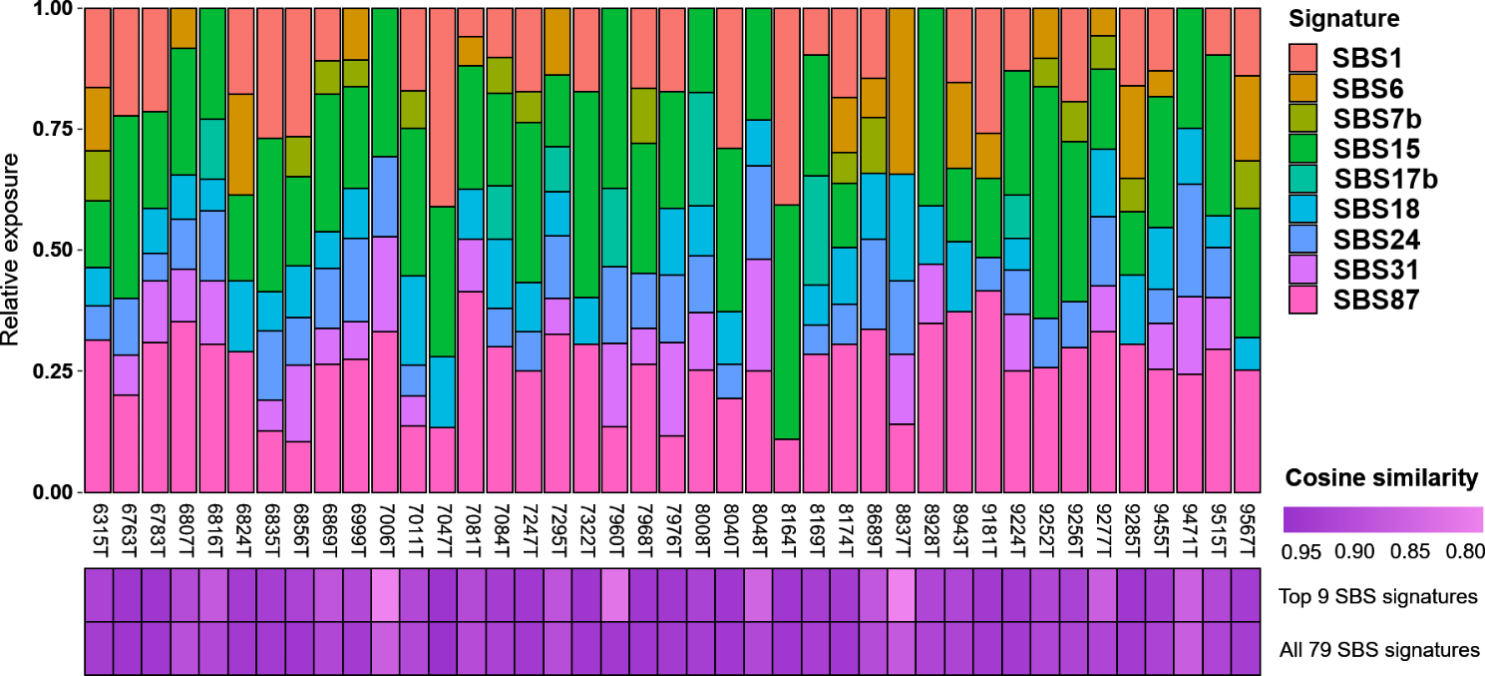
Relative contribution of top mutational signatures in mCLM. Top – relative exposures of top 9 SBS signatures in each sample. Bottom: top_SBSs – cosine similarity of the mutational catalog reconstructed from 9 most dominant signatures (above) to the original data. full_COSMIC_v3.3 – cosine similarity when fitting the full set of 79 COSMIC reference signatures (version 3.3). Few samples showed a substantial decrease in fitting accuracy by reduction of the number of signatures from 79 to 9

Next, relative contributions of reference SBS mutational signatures (COSMIC database v3.3) were determined for each mCLM sample. All 79 available reference SBSs were assessed in the first round (**Supplementary Table S5**). The top 10 signatures by overall contribution (in the whole cohort) were refitted to get their final relative contributions, except for SBS54, which was excluded due to being a suspected sequencing artefact by COSMIC. Reducing the mutational catalog to 9 signatures (Fig. 3, **Supplementary Table S6**) led to a negligible decrease in fitting accuracy in the vast majority of samples, as demonstrated by cosine similarity of the original mutational catalogs and the reconstructed catalogs from the top 9 signatures and from all 79 reference signatures (Fig. 3). A high correlation (r2 > 0.5, *p* < 0.001) between signature pairs SBS1-SBS15 (negative correlation) and SBS24-SBS31 (positive) in mCLM was observed.

### Germline profile of non-malignant liver samples

**Fig. 4:**
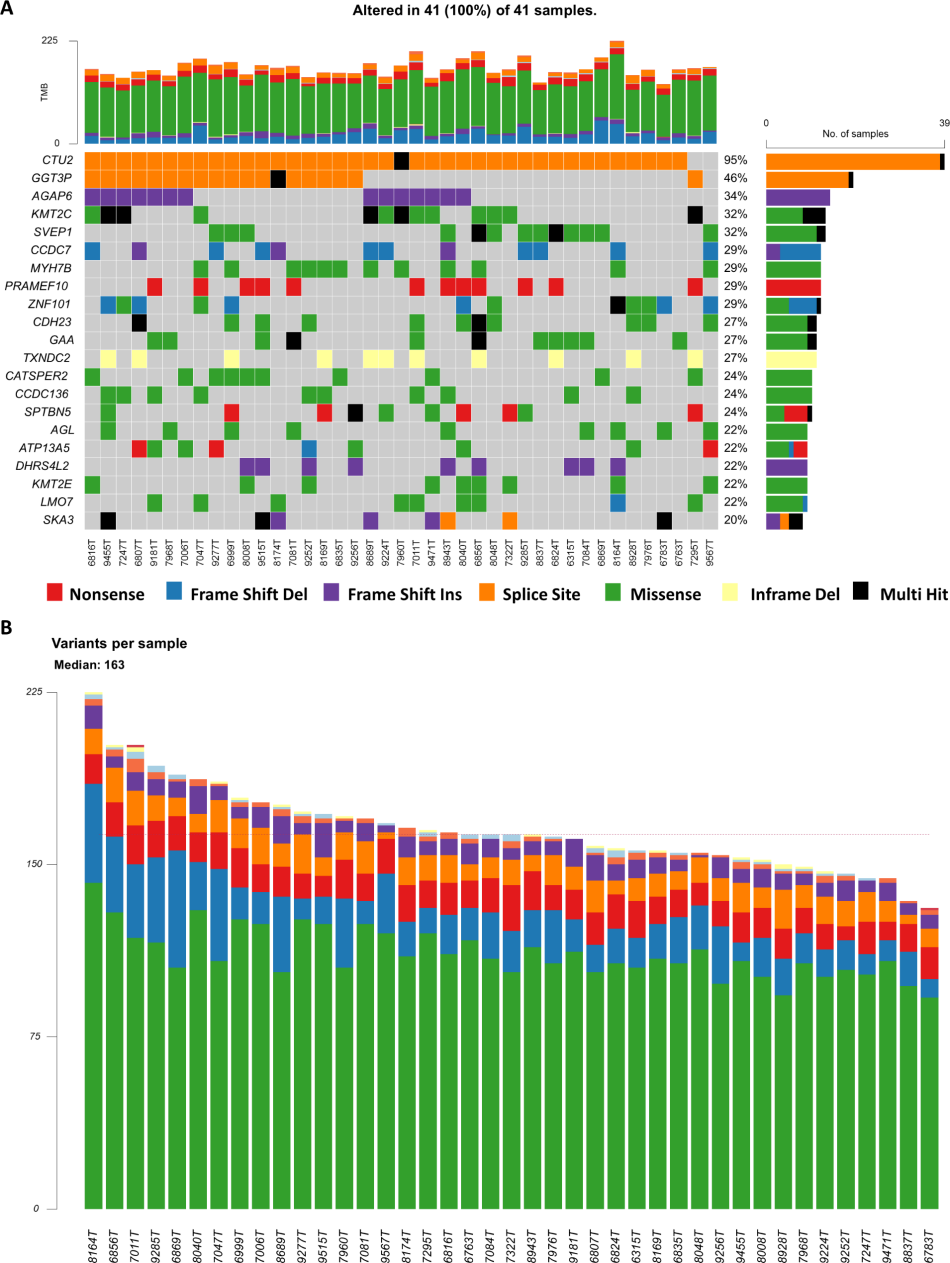
Distribution of rare and deleterious germline variants in non-malignant liver of mCLM patients. **(A)** plot depicting TOP20 most mutated genes fulfilling filtering conditions, **(B)** load of germline variants (median 163)

We performed the analysis of rare (allele frequency in gnomAD < 0.05) and deleterious (stop-gain, stop-loss, frameshift insertion or deletion, and changing the splice site or transcription start site predicted by VEP or listed as pathogenic in ClinVar or CADD score > 25) germline variants similarly to mCLM. In total, we found 5 573 variants. The median count was 184 (140–248) per patient. The median count of non-silent variants per patient was 163 and the most frequently mutated genes were *CTU2*, *GGT3P*, and *AGAP6* (Fig. 4A, B, **Supplementary Table S7**). Interestingly, these and some other genes, e.g., *CCDC7*, *PRAMEF10*, *ZNF101*, *SPTBN5*, or *DHRS4L2* had a high rate of variants with predicted HIGH functional effect (lollipops in **Supplementary Fig.****S3**). In the case of *CTU2*, all samples had the same variant (rs11278302) with a HIGH functional effect (splice donor variant) predicted. Due to its high frequency (95%), it was further not considered clinically relevant.

We also provide co-occurrence analysis of altered genes, together with somatic mutations. Except for already depicted interactions between somatic variants (**Supplementary Fig. ****S2**), germline variants in *AGAP6* or *ZNF101* co-occurred with somatic mutations in *KRAS* (*p* = 0.048 and *p* = 0.045, respectively) while *CATSPER2* was mutually exclusive (*p* = 0.002). *MOL7* was mutually exclusive to *APC* (*p* = 0.042). Germline variants in several genes co-occurred mutually too, e.g., *CDH23* and *ZNF101* (*p* = 0.007) (**Supplementary Fig.****S4**).

### Clinical relevance of germline and somatic profiles of the patients

Although there is no clear consensus about the definition of early recurrence [27], we divided patients by the 6 months RFS cut-off [28] to provide surrogate information about poor response to mCLM therapy. At the time of analysis, for 12 patients the relapse appeared within six months after curative surgery. Patients in this subgroup, had notably more variants in *ABCA13* and *UNC8*0, and fewer in *APC* and *RYR1* (**Supplementary Fig.****S5A**) but none of these differences were statistically significant. Moreover, patients with short RFS seemed to have exclusively nonsense variants in the *TP53* tetramer domain (*n* = 2 vs. none in long RFS, **Supplementary Fig.****S5B**), although this could be by chance and should be subject to validation using larger datasets. Stratification of patients according to subsequent systemic therapy after mCLM resection was not possible due to high heterogeneity.

Of the whole sample set, only two patients, 8164T and 7960T, were classified as TMB-high and had considerably high MSI status; however, only 7960T was classified MSI-high based on the strict 20% cut-off. Both had the MMR-D status using their somatic mCLM profile. One patient suffered from early recurrence and died six months after surgery. The other had OS of 82 months without recurrence signs by the censoring time point. Thus, these characteristics do not seem to provide prognostic information in our patient set.

Further, we analyzed prognostic associations of somatic variants in frequently altered genes and enrichment of any of the thirteen oncogenic pathways previously identified in CRC [29]. For this comparison, we used separately patients with variants classified as having a HIGH/MODERATE, or exclusively HIGH predicted functional effect against those without such variants. One patient was excluded from both RFS and OS analyses as lost to follow-up and two other patients were excluded from RFS analyses due to lung metastasis and absence of relapse-free period. Thus, 38 patients entered RFS and 40 OS analyses.

**Fig. 5:**
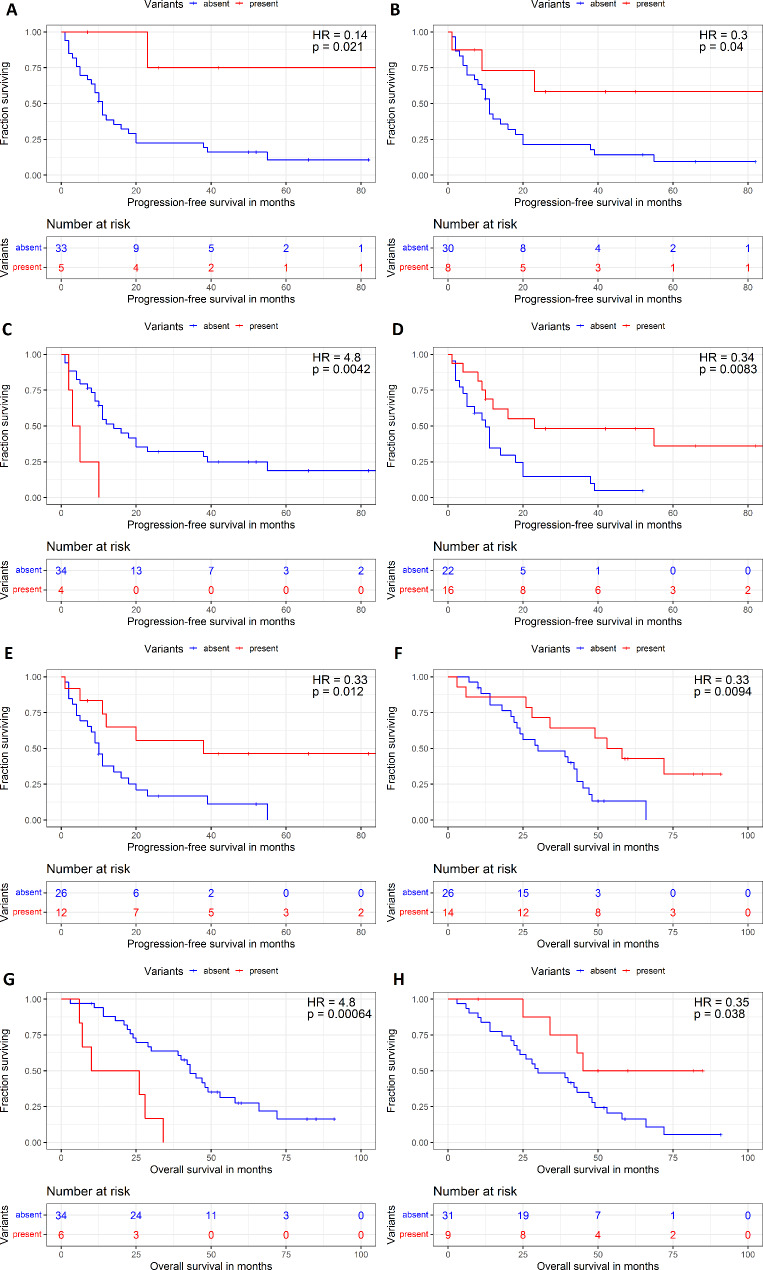
Kaplan-Meier plots of patient survival stratified by the carriage of variants in HRR, oncodriver pathways, and individual genes. **(A)** RFS analysis of somatic variants with HIGH functional effect in the HRR gene panel [30]. **(B)** RFS analysis of **(A)** complemented with rare pathogenic HRR variants. RFS analyses of somatic variants with HIGH or MODERATE functional effect in the MYC **(C)**, Notch **(D)**, and Hedgehog **(E)** pathways. OS analysis of somatic variants with HIGH or MODERATE functional effect in the JAK-STAT pathway **(F)**, *VIPR2***(G)**, and *MUC16* (**H**). Red line represents patients carrying the variant, and the blue line those without. HR = hazard risk

Most importantly, patients with HIGH functional effect only somatic variants in homologous recombination repair (HRR) genes [30] (*n* = 5; one in *ATR*, *BRCA2*, *NBN*, and two in *BRCA1*, **Supplementary Table****S8**) had significantly prolonged RFS compared to those without such variants (*p* = 0.021, Fig. 5A). Including patients with pathogenic germline variants (two in *ATM* and one in *RAD51C*) showed the same trend for RFS (*n* = 8, *p* = 0.040, Fig. 5B). OS analysis was not significant (*p* = 0.903 and 0.400, respectively). Concerning MMR genes (*MLH1, MLH3, MSH2, MSH3, MSH6*, and *PMS2* [31]), five patients had either somatic profile/HIGH functional effect variant (*n* = 3, two with TMB-H status and one with *MSH3* variant) or pathogenic germline variants (*n* = 2, both in *MLH1*). However, no significant prognostic association was observed.

Patients with HIGH or MODERATE functional effect somatic variants in the MYC pathway (*n* = 4, *MYC*, *MGA*, *MLXIP*, and *MLX*) had significantly worse RFS (*p* = 0.004, Fig. 5C), and those with somatically altered genes in the Notch (*n* = 16) or Hedgehog (*n* = 13) pathways had prolonged RFS (*p* = 0.008 and 0.012, respectively, Fig. 5D, E). Again, these associations were non-significant in OS analyses (*p* > 0.05). Importantly, patients with somatic variants in the JAK-STAT pathway (*n* = 14) had significantly prolonged OS (*p* = 0.009, Fig. 5F), but no association with RFS was found. The list of followed genes in pathways is in **Supplementary Table****S9**.

Regarding individual genes, patients carrying the somatic *KRAS* G12D variant (*n* = 6) had significantly poorer RFS compared to the rest of the patients (*p* = 0.044, **Supplementary Fig.****S6A**). This association was supported by the OS analysis of an external dataset (MSK panel data, *n* = 97) resulting in the same trend, i.e. shortened OS for patients with *KRAS* G12D variant compared to the rest (*p* = 0.020, **Supplementary Fig.****S6B**). No association was observed in patients stratified to wild type only vs. G12D variant (*p* > 0.05). *APC*, *TP53, FAT4, PIK3CA, FBXW7*, or other frequently mutated genes listed in the Cancer Gene Census (https://cancer.sanger.ac.uk/census) were not prognostic individually. However, patients bearing somatic variants in *VIPR2* (*n* = 6, missense) had significantly poorer OS than patients without such variants (*p* < 0.001, Fig. 5G). On the other hand, patients with somatic variants in *MUC16* (*n* = 8, one nonsense, and seven missense variants), encoding the CA125 tumor antigen, had significantly prolonged OS (*p* = 0.038, Fig. 5H). No patient had pathogenic germline or HIGH/MODERATE functional effect somatic variants in oncodrivers *BRAF* or *NRAS* that would be informative about the therapy. One patient had a somatic variant in *POLE*, together with *STK11*, one in *PTEN*, one in *MUTYH*, and two in *SMAD4.*

We then performed survival analyses of patients stratified using the TMB divided by the median and CNV number, size, and types. None of them was significant.

Next, survival analyses were done with top 9 SBS signatures divided by the median relative contribution but again no significant association was found.

Finally, we analyzed the prognostic significance of rare and deleterious germline variants in genes listed in Fig. 4A. Out of 19 individual genes and 13 oncodriver pathways fulfilling the above conditions (**Supplementary Table****S9**), only two were prognostic. Patients with alterations in *CCDC7* (*n* = 11) had significantly poorer RFS compared to wild-type carriers (*p* = 0.040, **Supplementary Fig.****S7A**) and similarly, patients with wild-type *KMT2E* (*n* = 31) had significantly worse OS than variant carriers (*p* = 0.017, **Supplementary Fig.****S7B**). Six patients had germline frameshift deletion p.Lys1250Aspfs*8 (rs371466318) in *CCDC7*, while the rest of the patients with alterations had frameshift insertions (*n* = 2, p.Leu77Phefs*6, rs146679927), or other deletions (*n* = 2, p.Asn919Leufs*11, rs202220321 and one novel p.Asn861Ilefs*21). The most frequent alteration did not influence RFS or OS. None of the *CCDC7* variants were reported in ClinVar. For *KMT2E*, all patients had the missense variant pGly999Cys (rs117986340), deleterious according to the SIFT predictor but benign according to ClinVar.

## Discussion

The mechanism of CRC metastasis is not fully understood. Liver metastasis is the most common site of secondary infiltration and metastatic growth followed by lung, lymph nodes, and peritoneum. Liver metastasis occurs in about 70% of all metastasizing colon and rectal cancers, and it is the most frequent site of solitary metastatic spread (48% of colon and 45% of rectal cancers) [32]. The treatment of metastatic CRC is mainly based on systemic therapy with regimens including 5-fluorouracil with oxaliplatin (FOLFOX, CAPOX), or irinotecan (FOLFIRI, CAPIRI). Based on the tumor *RAS/BRAF* mutational status, therapies involving anti-EGFR monoclonal antibodies (cetuximab, panitumumab) can be used. Patients with microsatellite instability-high (MSI-H) tumors or mismatch repair deficiency (MMR-D) can be treated with immunotherapy based on immune checkpoint inhibitors (ICIs) [3]. Despite this, reliable biomarker for the precision treatment is still missing. In addition, differences in metastatic scenario depending on the timing (i.e. synchronous vs. metachronous) are often not taken in consideration. Therefore, we describe the genetic background of the mCLM and establish candidate biomarkers of patient prognosis.

**Fig. 6:**
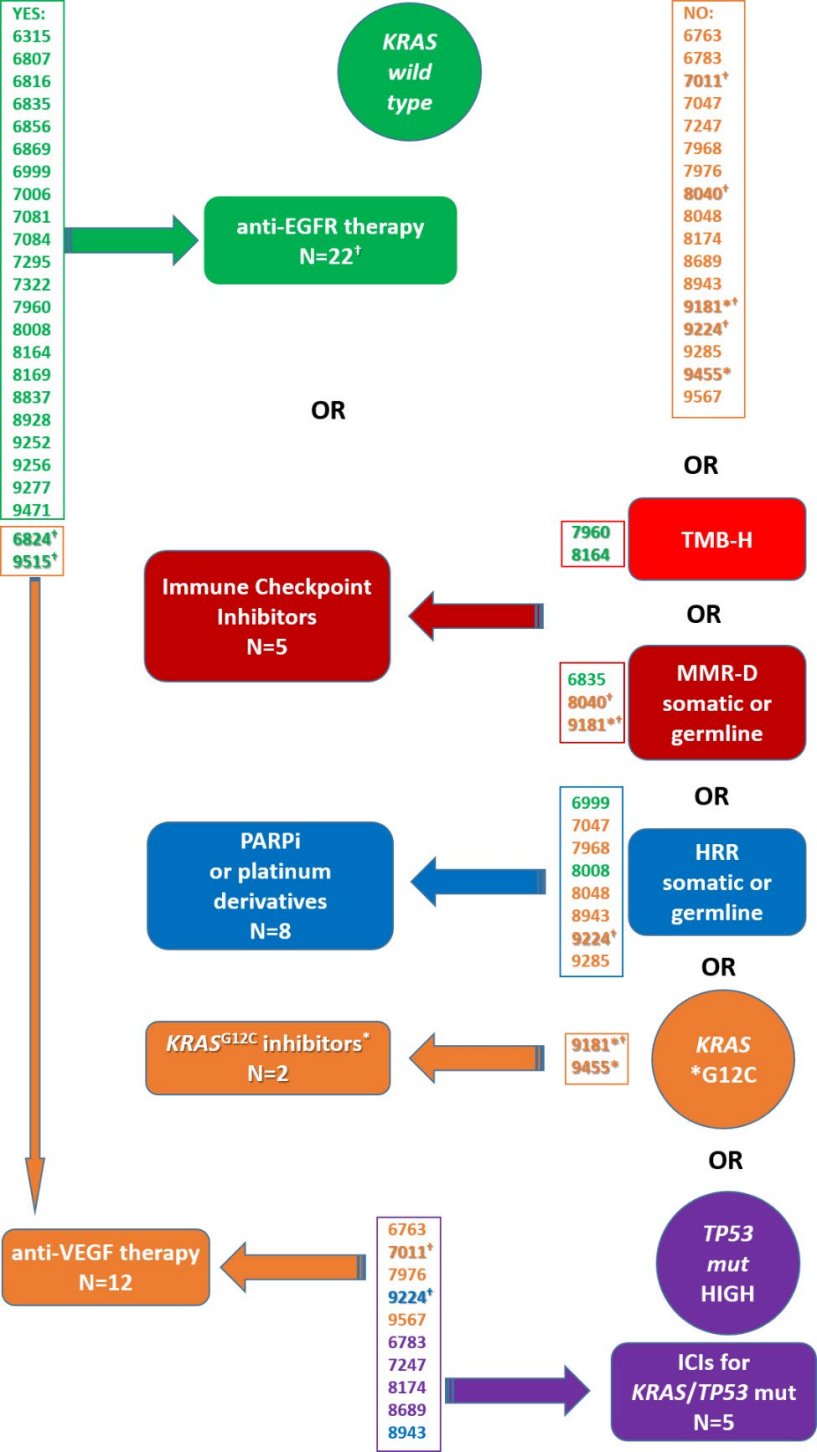
Diagram with proposed stratification of patients into targeted therapeutic regimens according to somatic and germline mCLM profiles. Numbers represent IDs of individual patients: TMB-H patients in light red, patients with MMR-D status in dark red, HRR in blue, *KRAS* wild type in green, *KRAS* mutated in orange (*KRAS^G12C^), and *TP53* mutated in violet. *KRAS* wild type patients eligible for the anti-EGFR therapy in the left column and the rest of patients with targetable mutations in the right column. Some patients belong to more groups based on their mutational profiles. Footnotes: ^*^Patients with somatic *KRAS*^G12C^ variant (*n* = 2). ^†^Patients with somatic variants in *PIK3CA* (*n* = 6; three p.E545K, one p.R93W, p.N345K, and p.Q546K) pathogenic or likely pathogenic by ClinVar

Firstly, according to our data, all patients can be stratified to categories used for choice of targeted therapy regimens in diverse cancers (Fig. 6). Based on analysis of *RAS/BRAF*, MMR-D, HRR, or gene mutational status, these patients may be offered treatments with immune checkpoint inhibitors (*n* = 5; ICI, e.g., nivolumab, atezolizumab, ipilimumab [33]), poly(ADP-ribose) polymerase inhibitors (*n* = 8; PARPi, e.g., olaparib, rucaparib [34]), or *KRAS* G12C specific inhibitors (*n* = 2; sotorasib, adagrasib [35]). Patients with *KRAS* and *PIK3CA* wild-type tumors (*n* = 22) may benefit from the anti-EGFR therapy (cetuximab, panitumumab), although for some portion of these patients, according to their genetic profiles, ICI or PARPi are an option as well. Two out of six patients with pathogenic somatic variants in *PIK3CA* had no other mutation that would enable stratification into one of the above groups, and thus, the anti-VEGF therapy remains the only option for them now. The rest of the patients with *KRAS* or *PIK3CA* mutated tumors are candidates for the anti-VEGF therapy too. These patients shall soon become eligible for clinical trials on inhibitors targeting frequent *KRAS* mutations, e.g., MRTX1133 for G12D or JAB-23,000 for G12V [36]. Similarly, the *TP53* mutated fraction of patients (*n* = 10) constitutes a group that may enter future clinical trials on diverse p53 targeting strategies [37]. In light of recent reports, these patients may also be considered for ICIs [38]. In line with this, a subgroup of patients with *KRAS* and *TP53* mutually mutated tumors, especially those with variants having the HIGH functional effect prediction for *TP53* (*n* = 5), may benefit from anti-PD-L1 therapy, e.g., with atezolizumab, combined with bevacizumab and chemotherapy as recently demonstrated in non-small-cell lung cancer [39]. We are aware that some of the above treatments are under development or so far reserved for other cancer diagnoses and thus our assumptions should be taken as inspiration for further clinical trials plans. It seems that the selection of patients for the above-mentioned targeted therapeutic options may require quite a small gene panel. Future studies involving different metastatic scenarios and tissue specimens from more than single loci should determine which portion of patients may benefit from such an approach and for which a global (exome/genome) approach and eventually other omics may be necessary.

The most commonly somatically mutated genes in mCLM were *APC*, *TP53*, and *KRAS*. This observation complies with the TCGA dataset COAD-READ and published data on primary [7] and metastatic [11] tissues.

The detailed statistical evaluation of the results revealed several associations providing prognostic information. Similarly as in patients with ovarian carcinoma [40], patients with HIGH functional effect somatic or pathogenic germline variants in HRR genes had prolonged RFS without effect on OS. Thus, the HRR pathway seems to have a general prognostic value in solid cancers. Additionally, patients with HIGH or MODERATE functional effect somatic variants in the MYC pathway had significantly worse RFS in compliance with the recently reported negative prognostic impact of mutations in this oncodriver pathway [41]. In contrast, patients with somatically altered genes in the Notch or Hedgehog oncodriver pathways had significantly prolonged RFS, in line with another report suggesting that inhibition of these pathways restores the chemosensitivity of in vitro CRC cell models and organoids [42]. Finally, patients with somatic variants in the JAK-STAT pathway had prolonged OS, likewise supporting a previous observation of the oncosuppressive potential of its inhibition [43].

Among individual genes, patients carrying the somatic *KRAS* G12D variant had significantly poorer RFS compared to the rest of the patients (wild type or different variants). We recently reported that patients with synchronous CRC metastases, harboring *KRAS* variants in primary tumors, had poor relapse-free survival, and this association was confirmed in the TCGA COAD-READ dataset [13], thus corroborating previous reports [44]. Analysis of external dataset containing 97 mCLM patients confirmed the present study results suggesting that *KRAS* 12D variant has general prognostic role in metastatic CRC. Patients with somatic variants in *MUC16* had significantly prolonged OS. It is worth mentioning that this gene encodes the CA125 tumor antigen [45] and is hence relevant to precision oncology. Finally, the strongly negative prognostic value of *VIPR2* variants towards OS (*p* < 0.001) represents a novel observation. *VIPR2* encodes the vasoactive intestinal peptide receptor 2, a G protein-coupled receptor that functions as a neurotransmitter and a neuroendocrine hormone [46], with recently reported diagnostic relevance for CRC [47]. The analysis of protein-protein interaction networks by the STRING tool (https://string-db.org/) shows high confidence (> 0.9) links between VIPR2 and several members of the G protein family (VIPR1, FSHB, GNAS, and GNB1) and adenylate cyclase-activating polypeptide 1 (ADCYAP1), which promotes neuron projection development through the RAS/ERK pathway (**Supplementary Fig.****S8**). Interestingly, a most recent report suggested that VIPR2 blockade by a specific inhibitor has anticancer effects both as a monotherapy and in combination with ICIs [48]. Future functional studies should help better understand these observations. Due to the lack of suitable datasets for validation, a conclusive assessment is impossible, and thus, our results must be interpreted with extreme caution.

Our study has several limitations and benefits. Although the sample size may seem small, we report the most extensive analysis on mCLM to date using fresh frozen tumor material. More commonly, tissues are available as formalin-fixed paraffin-embedded (FFPE) blocks, which represents a substantially lower quality source of DNA and results in less reliable, more error-prone, and often significantly biased data. We exploited that our center specializes in treatment of CRC liver metastases and thus can assemble a high-quality sample set of a relatively large cohort of patients treated and followed up homogeneously. We considered increasing the size by including synchronous metastases but refused to do it due to the reported differences in prognosis and therapy of these two metastatic scenarios [4]. Despite this effort, heterogeneity of administered adjuvant and palliative treatment after mCLM resection prevented detailed analyses. The lack of primary tumor tissues, which would enable the study of genetic changes during tumor progression with potential therapeutic consequences, poses another limitation. However, the relatively long time period between the surgical treatment of primary and metastatic disease resulted in the fact that in some cases the former procedure was performed at a different center than the latter, and primary tissue was not available at all or in the FFPE form, which could introduce bias. Next, various post-operative adjuvant chemotherapies were administered after hepatectomy including treatments that are not recognized as standard care. New therapeutics and novel treatment strategies may come out in the future, however, at the present state this fact limits our study. Finally, we acknowledge serious limitations connected with the lack of validation of our data using external datasets. We searched the TCGA COAD-READ dataset, but the vast majority of available data comes from primary tissues, and for metastatic loci, no information to distinguish between synchronous and metachronous metastasis scenarios is available. For the targeted panel sequenced, the AACR GENIE cohort [49], the metastatic site is not specified (v13.1-public; 65.6% “unspecified”, 29.7% “distant organ”, 2.8% “lymph node”, and 1.9% “local recurrence”) and comparable survival data is not publicly provided. Finally, the MSK dataset was useful only for confirmation of the *KRAS* 12D prognostic value because this panel did not contain *VIPR2* and *MUC16*, and genes from the oncogenic pathways were seriously underrepresented too, precluding their validation. Therefore, we consider it necessary for future meta-analyses and validations to provide additional genomic profiles with robust sequence coverage accompanied by complete clinical follow-up.

In conclusion, we report new putative prognostic biomarkers of mCLM and demonstrate that a relatively small number of genes is informative about the available, or soon upcoming, targeted therapies for eventually relapsing patients after radical mCLM surgery. These results underscore the recent recommendation of the ESMO Precision Medicine Working Group towards genetic screening of metastatic colorectal cancer by clinical research centers for stratifying patients to clinical trials and accelerating drug development [50], encourage further elucidation of the molecular background of patients with CRC liver metastases, and provide additional data for the concept of personalized therapy.

### Electronic supplementary material

Below is the link to the electronic supplementary material.

Supplementary Material 1

Supplementary Material 2

## Data Availability

All data generated or analyzed during this study are included in this published article and its supplementary information files. Sequencing data aligned to the canonical hg38 reference genome (BAM files) were submitted to the Sequence Read Archive (SRA) under the BioProject ID: PRJNA896777 (https://www.ncbi.nlm.nih.gov/sra/PRJNA896777).

## List of abbreviations

BAF: B–allele frequencies
BWA: Burrows–Wheeler Aligner
CLM: Colorectal liver metastases
CNVs: Copy number variants
CRC: Colorectal cancer
FFPE: Formalin–fixed paraffin–embedded
FLAGS: FrequentLy mutAted GeneS
GATK: Genome Analysis Toolkit
HGNC: Human Genome Organisation Gene Nomenclature Committee
HRR: Homologous recombination repair
indels: Small insertions–deletions
MAF: Mutation annotation format
mCLM: Metachronous colorectal liver metastases
MEM: Maximal exact matches
MMR: D–mismatch repair deficiency
MSI: H–microsatellite instability–high
OS: Overall survival
RFS: Relapse–free survival
SNV: single nucleotide variant
TMB: Tumor mutation burden
VEP: Variant Effect Predictor
