# Supplementary material for: Prognostic potential of whole exome sequencing in the clinical management of metachronous colorectal cancer liver metastases
Source: Cancer Cell Int. 2023 Nov 26;23:295. doi: 10.1186/s12935-023-03135-x (PMC10676609; doi:10.1186/s12935-023-03135-x)
Supplement: Supplementary file 1 — Supplementary Material 1 [file 12935_2023_3135_MOESM1_ESM.pdf]

Supplementary Figures

Prognostic potential of whole exome sequencing in the clinical management of metachronous colorectal cancer liver metastases

Heczko Lucie, Hlaváč Viktor, Holý Petr, Dvořák Pavel, Liška Václav, Vyčítal Ondřej, Fiala Ondřej, Souček Pavel

List of Supplementary Figures

Supplementary Figure S1: Lollipop plots of the topology of *APC* and *TP53* variants in mCLM
Supplementary Figure S2: The analysis of co-occurrence and mutual exclusivity of somatic variants among the most frequently altered genes in mCLM
Supplementary Figure S3: Lollipop plots of the topology of germline variants with predicted pathogenic effect genes with functional domains identified in non-malignant liver samples from mCLM patients
Supplementary Figure S4: The analysis of co-occurrence and mutual exclusivity of somatic and germline with predicted pathogenic effect variants in mCLM patients
Supplementary Figure S5: Oncoplot of patients stratified by progression-free survival with cut off 6 months
Supplementary Figure S6: Kaplan-Meier plot of survival of patients stratified by the carriage of somatic *KRAS* G12D variant
Supplementary Figure S7: Kaplan-Meier plots of patient survival stratified by the carriage of germline variants in non-malignant liver samples
Supplementary Figure S8: VIPR2 protein-protein interaction network according to the STRING database

Supplementary Figure S1: Lollipop plots of the topology of *APC* and *TP53* variants in mCLM (A) *APC*, (B) *KRAS*, (C) *TP53*

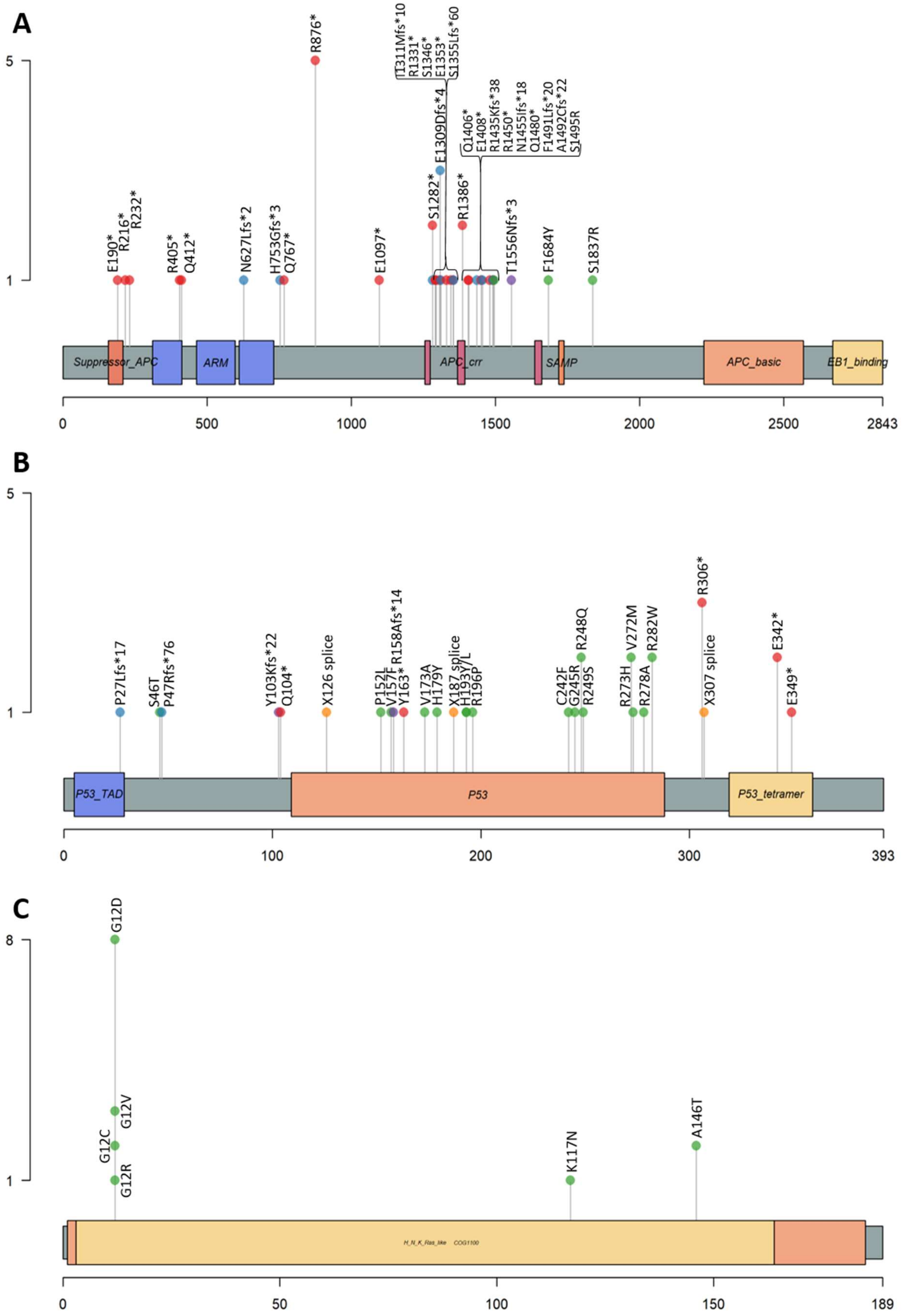

Supplementary Figure S2: The analysis of co-occurrence and mutual exclusivity of somatic variants among the most frequently altered genes in mCLM

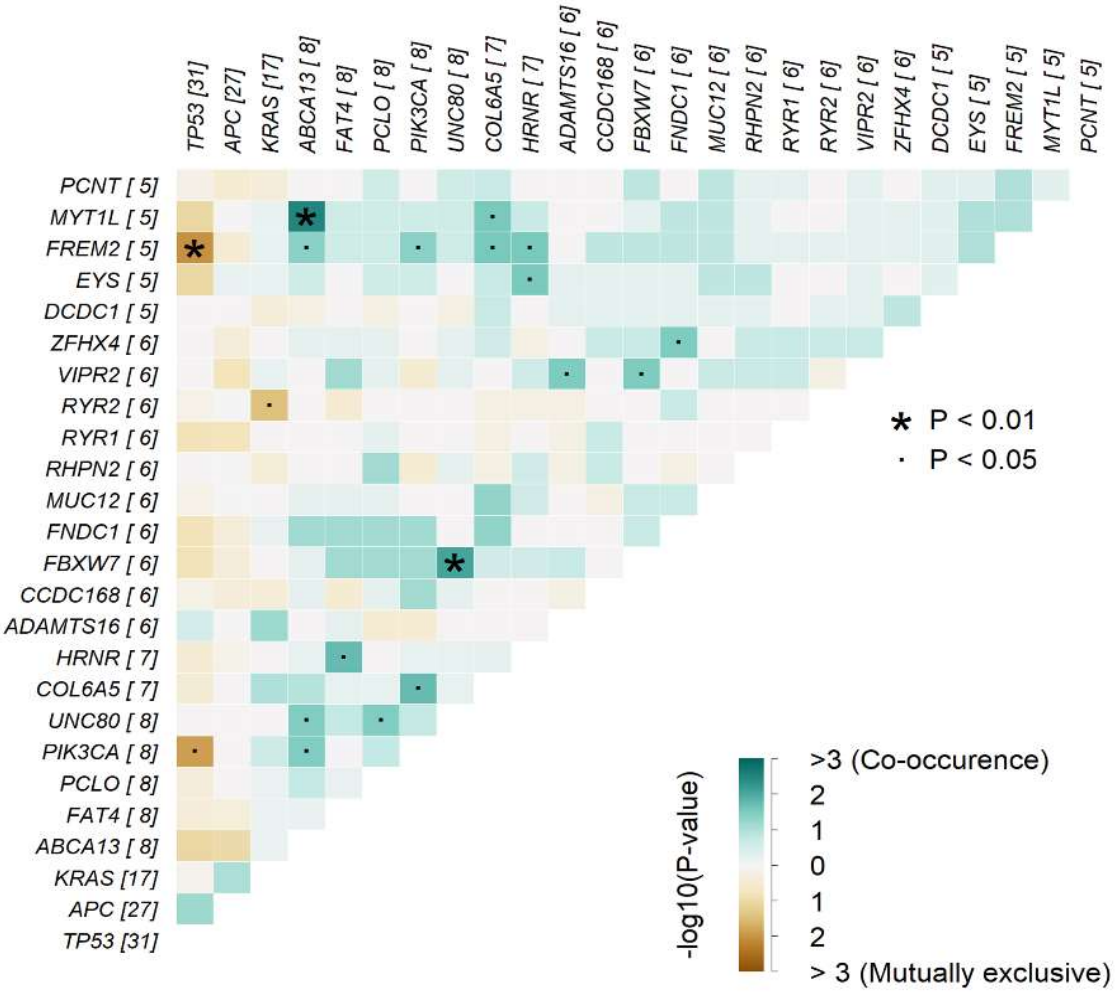

Supplementary Figure S3: Lollipop plots of the topology of germline variants with predicted pathogenic effect genes with functional domains identified in non-malignant liver samples from mCLM patients
(A) *CTU2,* (B) *AGAP6,* (C) *ZNF101,* (D) *SPTBN5,* and (E) *DHRS4L2*

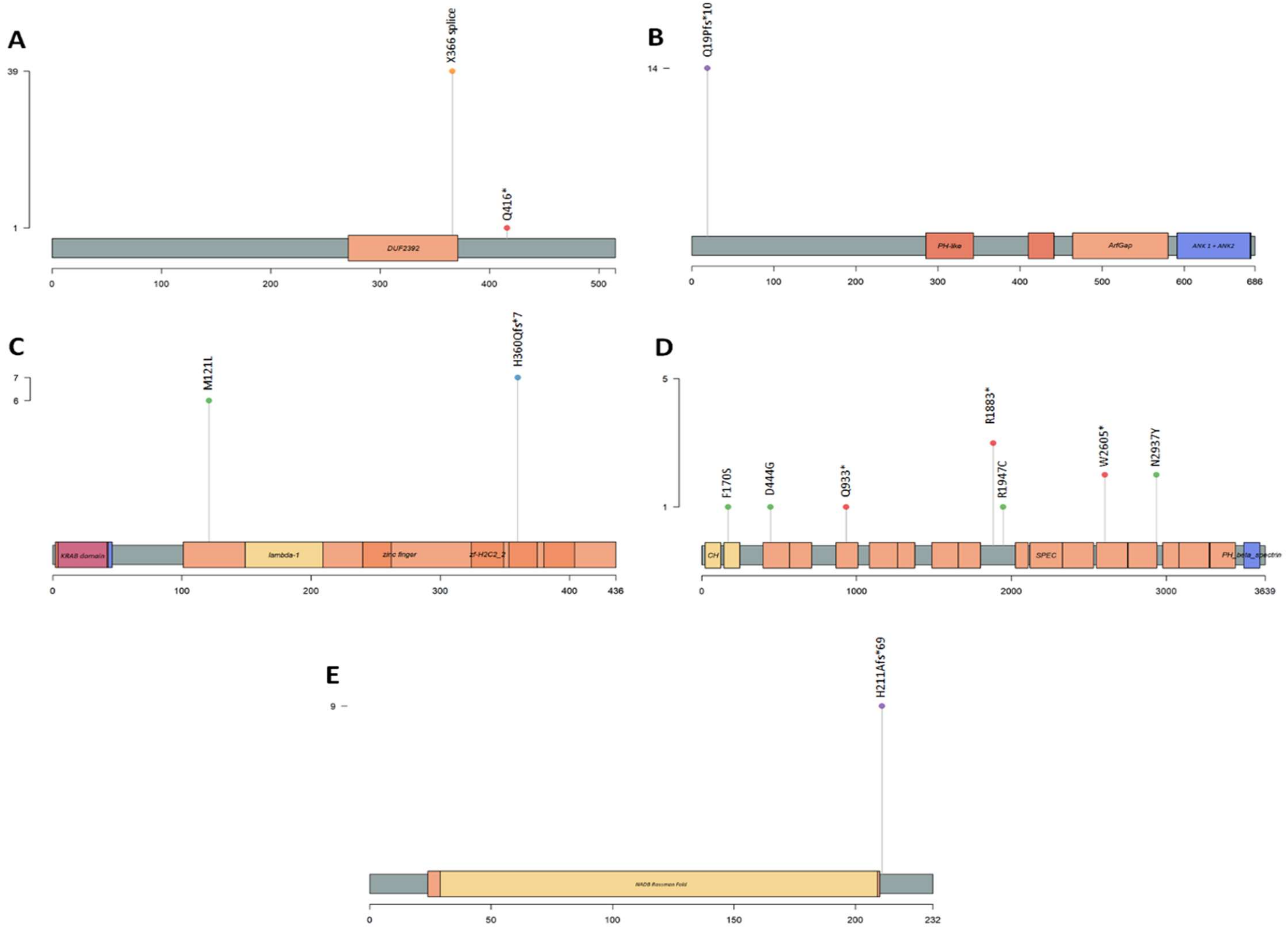

Supplementary Figure S4: The analysis of co-occurrence and mutual exclusivity of somatic and germline with predicted pathogenic effect variants in mCLM patients
The “g” in front of the gene symbol means germline; the rest of genes is somatically mutated.

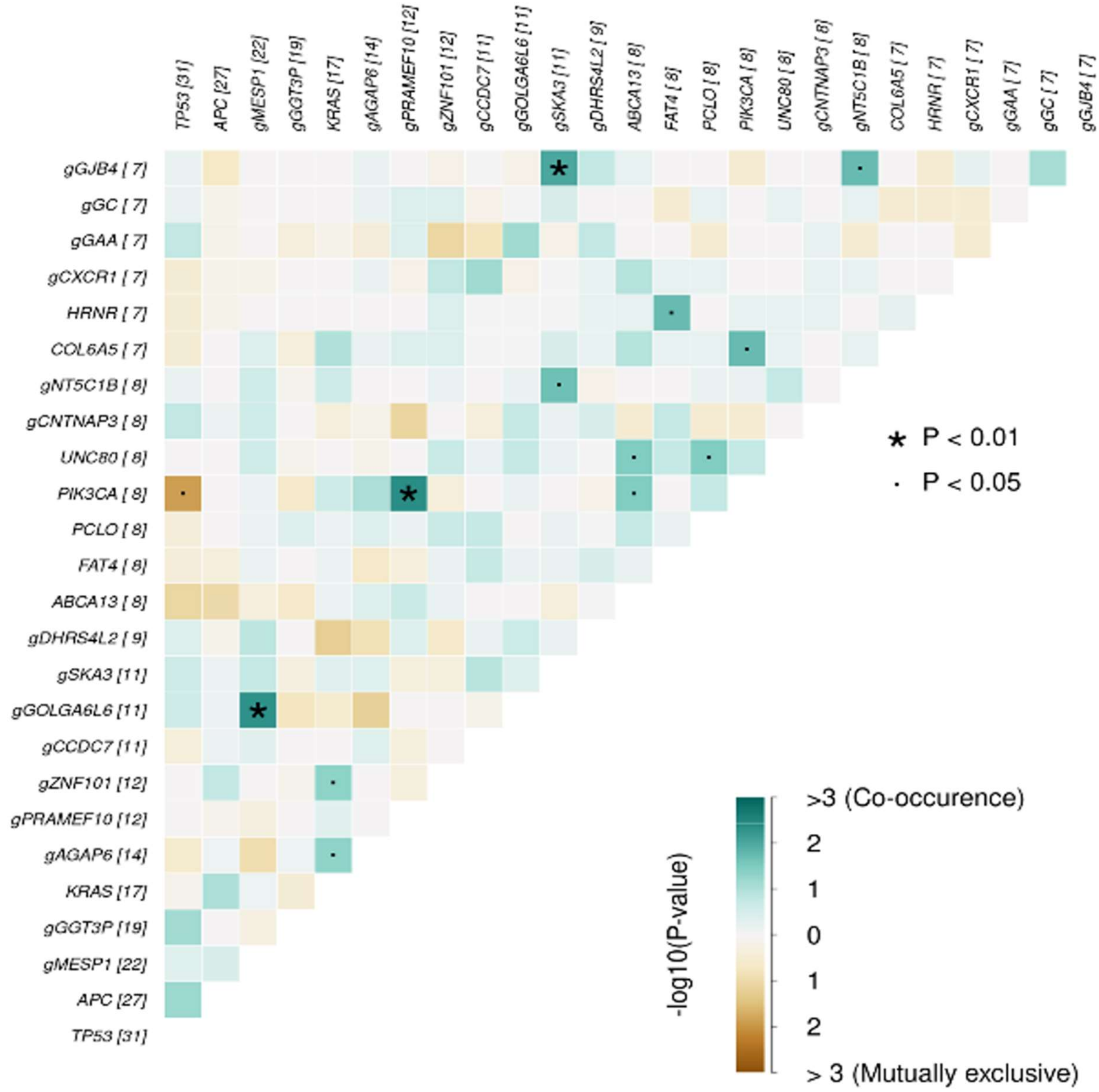

Supplementary Figure S5: Oncoplot of patients stratified by progression-free survival with cut off 6 months

(A) Oncoplot with the most frequently mutated genes, (B) lollipop plots of the topology of *APC* and *TP53* variants in mCLM of patients divided by the cut off PFS 6 months.

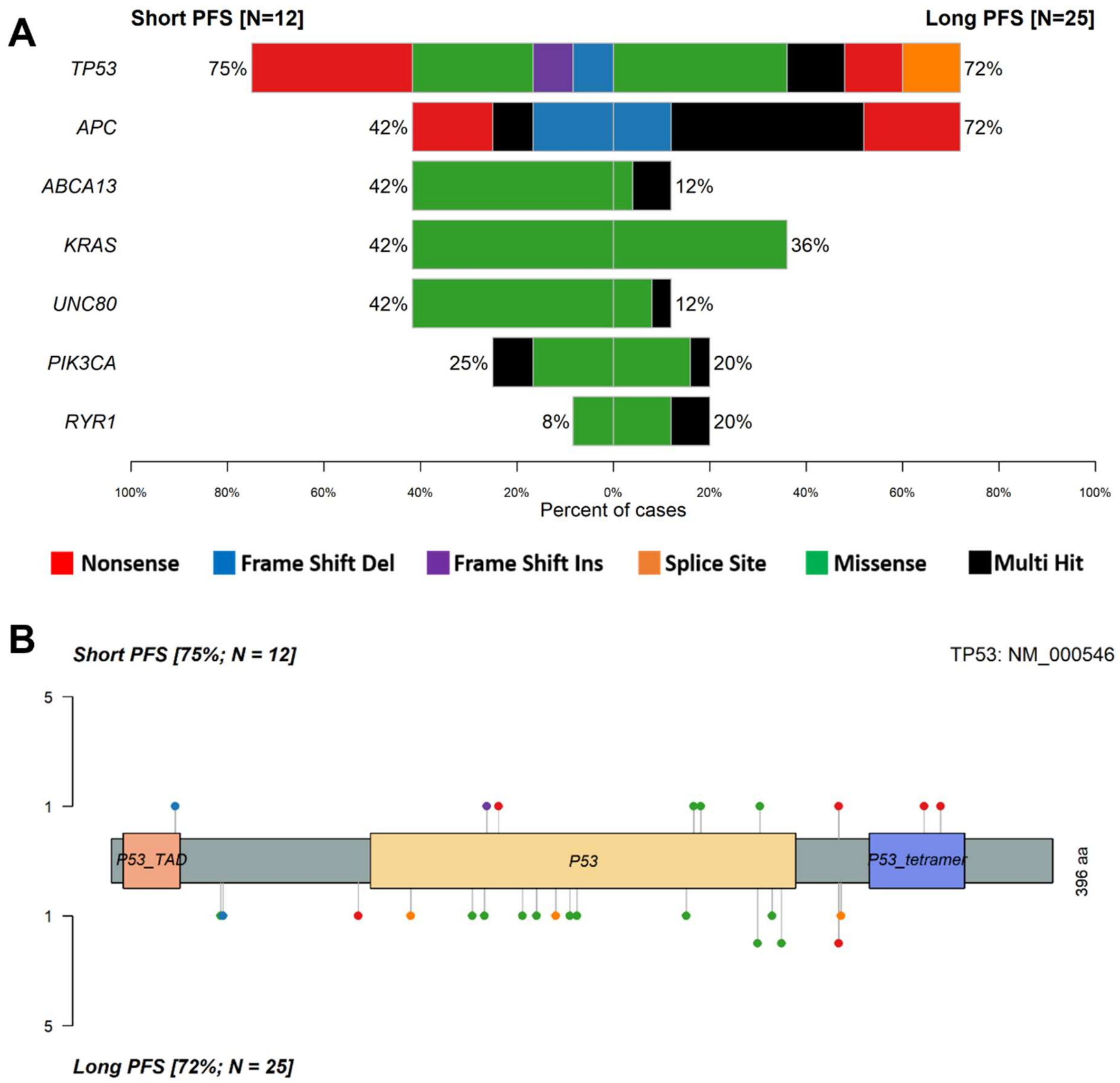

Supplementary Figure S6: Kaplan-Meier plot of survival of patients stratified by the carriage of somatic *KRAS* G12D variant
(A) RFS for patients with mCLM from the present study (n=38) and (B) OS for mCLM cohort from external MSK dataset (n=97) censored at 120 months. Red line represents patients carrying the variant, and the blue line those without.
HR=hazard risk

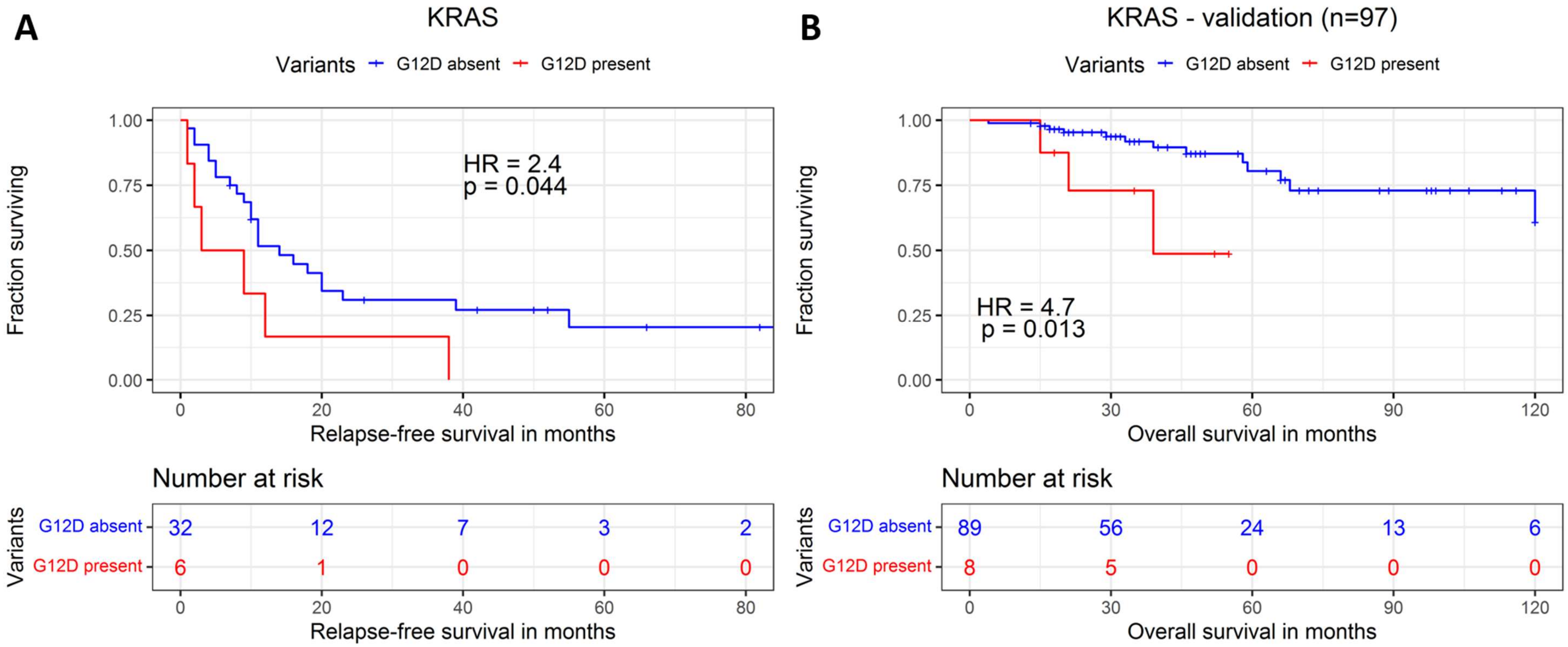

Supplementary Figure S7: Kaplan-Meier plots of patient survival stratified by the carriage of germline variants in non-malignant liver samples (A) RFS for patients stratified by carriage of variants in *CCDC7* and (B) OS for those stratified by variants in *KMT2E*
Red line represents patients carrying the variant, and the blue line those without.
HR=hazard risk

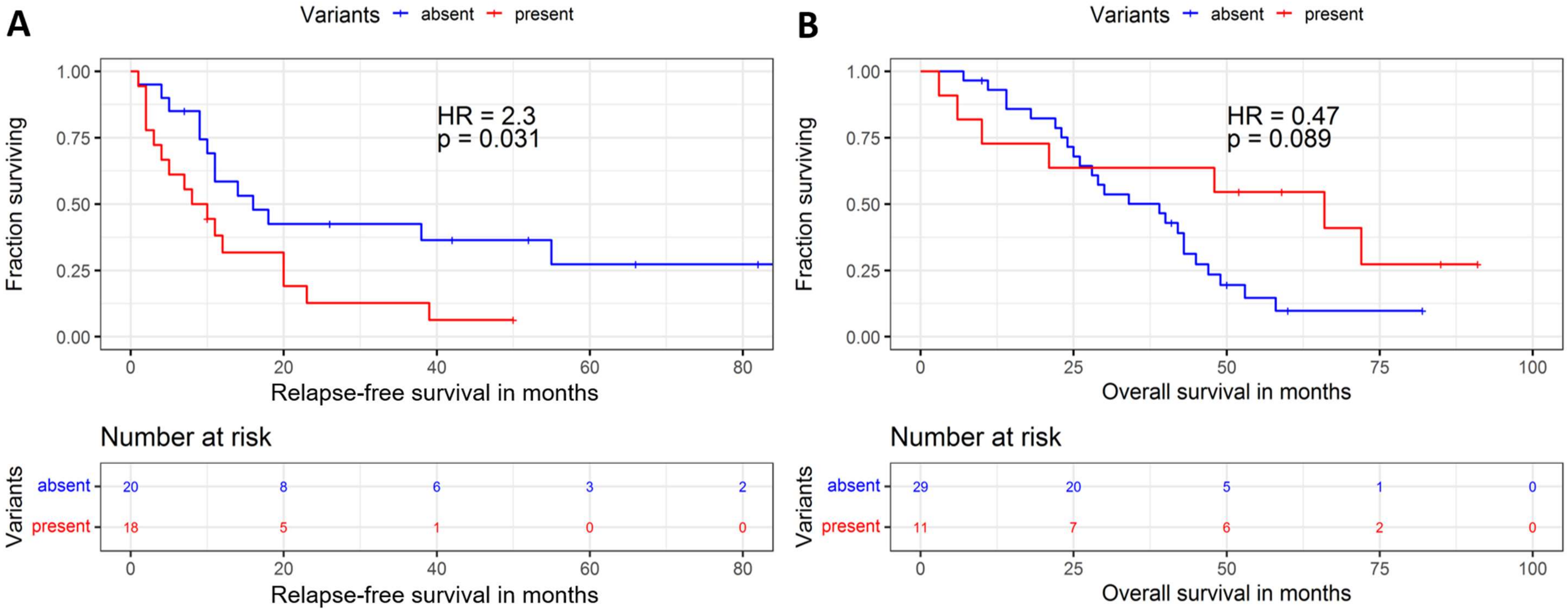

Supplementary Figure S8: VIPR2 protein-protein interaction network according to the STRING database (https://string-db.org/cgi/network)

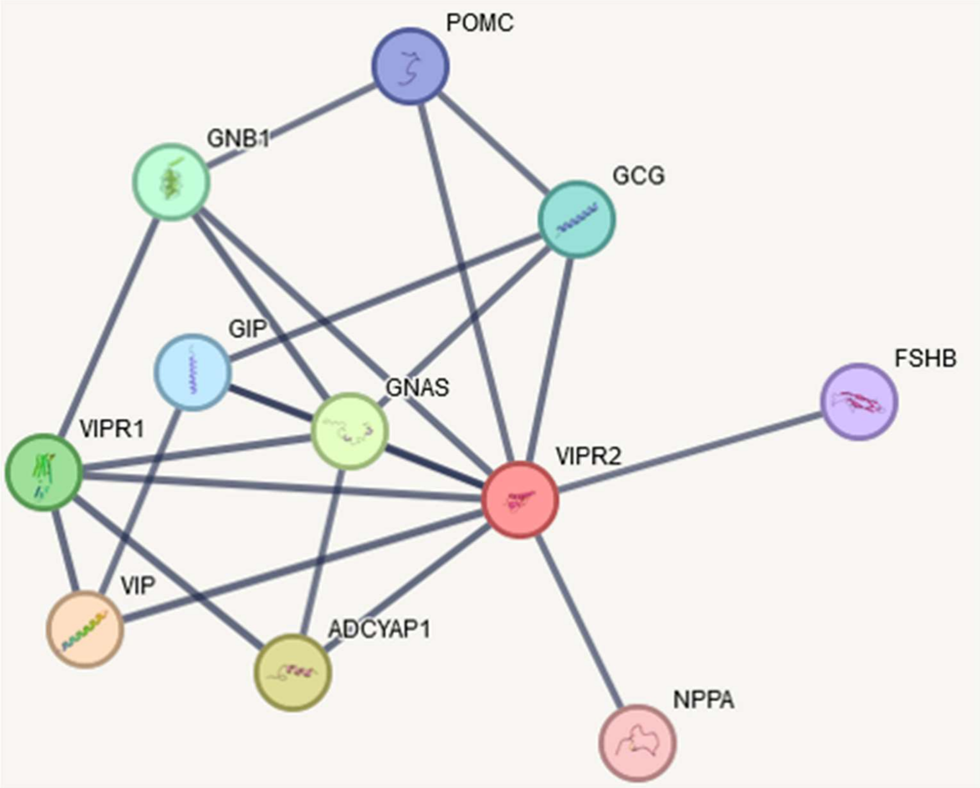

The network is based on the highest confidence (>0.9) limited to no more than 10 interactors.

G proteins and G protein-coupled receptors:
VIPR2 - Vasoactive intestinal peptide receptor 2
FSHB - Follicle-stimulating hormone, beta polypeptide
GNAS - Guanine nucleotide-binding protein G(s), alpha-stimulating activity polypeptide 1, included
GNB1 - Guanine nucleotide-binding protein, beta-1
VIPR1 - Vasoactive intestinal peptide receptor 1

Other proteins:
VIP - Vasoactive intestinal peptide
POMC - Proopiomelanocortin
GCG - Glucagon
NPPA - Natriuretic peptide precursor A
ADCYAP1 - Adenylate cyclase-activating polypeptide 1

KEGG pathways ($p<0.05$ after FDR): insulin and renin secretion, cAMP signaling, ovarian steroidogenesis, and regulation of lipolysis in adipocytes.
