# Supplementary material for: Prognostic potential of whole exome sequencing in the clinical management of metachronous colorectal cancer liver metastases
Source: Cancer Cell Int. 2023 Nov 26;23:295. doi: 10.1186/s12935-023-03135-x (PMC10676609; doi:10.1186/s12935-023-03135-x)
Supplement: Supplementary file 2 — Supplementary Material 2 [file 12935_2023_3135_MOESM2_ESM.pdf]

Supplementary Tables

Prognostic potential of whole exome sequencing in the clinical management of metachronous colorectal cancer liver metastases

Heczko Lucie, Hlaváč Viktor, Holý Petr, Dvořák Pavel, Liška Václav, Vyčítal Ondřej, Fiala Ondřej, Souček Pavel

Contents

Table S1: Clinical characteristics of mCLM patients
Table S2: Basic description of somatic variants including TMB, MSI, and MMR-D
Table S3: TOP 20 most frequently altered genes in mCLM
Table S4: Basic description of somatic copy number variations in mCLM
Table S5: Relative contributions all 79 reference SBS mutational signatures in COSMIC v3.3 (see Materials and Methods)
Table S6: Relative contributions of the top 10 most significant reference SBS mutation signatures (COSMIC v3.3, see Materials and Methods)
Table S7: TOP 20 most frequently altered genes in non-malignant liver samples
Table S8: Functionally relevant list of somatic and gemline variants in HRR and MMR genes in mCLM
Table S9: List of genes used for evaluation of somatic variants in oncodriver pathways in mCLM

Table S1: Clinical characteristics of mCLM patients

Parameter	Mean ± S.D.	Number of patients	Percentage
Age (years)	65.0 ± 8.5 (range 37-77)	41	100
Sex			
Male		24	58
Female		17	42
**Primary diagnosis**			
Primary tumor sideness			
Right side		10	24
Left side		31	76
*Tumor extent (pT)**			
pT1		0	0
pT2		9	22
pT3		29	73
pT4		2	5
*Regional lymph node involvement (pN)**			
pN0		19	48
pN1		17	42
pN2		4	10
*Distant metastasis (pM)**			
pM0		41	100
pM1		0	0
*TNM stage of the primary tumor according to AJCC 2018**			
Stage I		6	15
Stage IIA/IIB		13	32
Stage IIIA-C		21	53
Stage IV		0	0
Grade (G)			
G1		8	19
G2		20	50
G3		8	19
GX		5	12
Adjuvant chemotherapy or radiotherapy			
Administered†		32	22
Not administered		9	78
**Characteristics of metastases**			
Average time to first metastasis diagnosis (months)	27.0 ± 21.0 (range 6-95)		
Number of metastatic loci			
single		32	78
two		6	15
three or more		3 (range 3-5)	7
Average size of metastases (cm)	5.4 ± 3.4 (range 1-16)		
mCLM resection radicality			
Radical (pR0)		35	85
Non-radical (pR1)		6	15
Adjuvant chemotherapy after mCLM resection			
Administered†		23	56
Not administered		18	44
Progression-free survival after mCLM resection ‡			
≤6 months		12	32
>6 months or no progression		26	68
Overall survival (months) after mCLM resection #	40.0 ± 6.6 (range 3-103)	40	98

Footnotes:

*For one patient the pTNM stage was not available

†Regimen containing 5-fluorouracil, oxaliplatin, capecitabine with or without radiotherapy.

After mCLM resection, targeted therapy (Vectibix, Avastin or Stivarga) added to palliative regimen in seven patients.

‡Three patients with oligometastic process excluded from RFS analyses
#One patient early lost to follow-up excluded from OS analyses

Table S2: Basic description of somatic variants including TMB, MSI, and MMR-D in mCLM

Tumor_Sample_Barcode	HIGH predicted effects*						MODERATE predicted effects*			Total HIGH+MODERATE	Total All**	TMB (per MB)***	MSI****			MMR-D*****
	Frame_Shift_Del	Frame_Shift_Ins	Nonsense_Mutation	Nonstop_Mutation	Translation_Start_Site	Splice_Site	In_Frame_Del	In_Frame_Ins	Missense_Mutation				Valid_sites	MSI_sites	MSI_score(%)	
6315T	2	2	2	0	0	3	3	0	61	73	155	2.08	1530	22	1.44	0.37
6763T	2	1	3	0	0	1	3	0	94	104	252	2.96	1899	25	1.32	0.40
6783T	4	2	10	0	0	5	3	0	152	176	360	5.01	1505	21	1.40	0.68
6807T	5	4	3	0	1	2	3	1	89	108	245	3.08	1821	21	1.15	0.74
6816T	2	2	6	0	0	2	3	0	162	177	384	5.04	1284	14	1.09	0.37
6824T	3	2	7	0	0	4	2	0	105	123	269	3.50	1704	25	1.47	0.34
6835T	2	1	8	0	0	3	4	0	80	98	219	2.79	1812	26	1.43	0.43
6856T	1	0	5	0	0	2	0	2	99	109	216	3.11	1460	14	0.96	0.28
6869T	0	0	3	0	0	0	0	0	34	37	84	1.05	1353	19	1.40	0.09
6999T	8	3	17	0	1	8	7	2	222	268	560	7.64	1837	25	1.36	1.03
7006T	1	0	1	0	0	0	2	0	28	32	82	0.91	1783	28	1.57	0.20
7011T	3	3	9	0	0	1	3	0	111	130	317	3.70	1703	18	1.06	0.51
7047T	0	4	1	0	1	4	1	0	113	124	253	3.53	1632	29	1.78	0.23
7081T	4	1	9	1	1	1	9	0	129	155	316	4.42	1739	19	1.09	0.68
7084T	4	2	8	1	0	3	0	0	187	205	407	5.84	1850	25	1.35	0.37
7247T	2	3	8	0	0	5	1	0	163	182	389	5.19	1259	20	1.59	0.48
7295T	2	5	4	0	0	1	1	0	58	71	186	2.02	1820	17	0.93	0.34
7322T	2	4	6	0	0	3	1	0	95	111	222	3.16	1869	18	0.96	0.31
7960T	84	28	10	2	3	12	4	1	472	616	1441	17.55	1887	441	23.37	8.86
7968T	3	0	3	0	1	0	1	0	112	120	248	3.42	1764	16	0.91	0.23
7976T	6	5	10	1	0	1	0	0	114	137	295	3.90	1719	25	1.45	0.74
8008T	3	1	11	0	0	6	2	0	211	234	564	6.67	1651	13	0.79	0.83
8040T	4	1	9	0	0	1	0	0	141	156	320	4.44	1635	28	1.71	0.34
8048T	6	0	3	0	1	6	3	0	134	153	312	4.36	1699	21	1.24	0.66
8164T	157	43	51	2	0	15	15	1	1224	1508	2932	42.96	1785	194	10.87	12.14
8169T	3	4	7	0	0	5	2	1	223	245	545	6.98	1785	19	1.06	0.80
8174T	4	1	4	0	0	3	3	0	106	121	280	3.45	1630	22	1.35	0.34
8689T	0	0	4	0	0	3	3	0	67	77	173	2.19	1732	22	1.27	0.20
8837T	2	1	3	1	0	4	0	1	29	41	103	1.17	1730	19	1.10	0.37
8928T	1	0	4	0	0	8	3	5	130	151	323	4.30	1687	22	1.30	0.51
8943T	5	0	3	0	0	2	0	0	59	69	174	1.97	1659	22	1.33	0.34
9181T	6	2	9	0	0	2	1	1	150	171	367	4.87	1945	13	0.67	0.74
9224T	5	9	8	0	1	3	4	1	170	201	406	5.73	1425	17	1.19	0.97
9252T	1	2	2	0	0	0	4	0	43	52	106	1.48	1868	14	0.75	0.37
9256T	1	0	3	0	0	3	2	0	68	77	163	2.19	1566	22	1.40	0.20
9277T	3	3	10	0	0	4	3	1	127	151	333	4.30	1643	24	1.46	0.97
9285T	1	3	3	0	0	0	2	0	76	85	193	2.42	1738	15	0.86	0.31
9455T	2	0	7	0	0	1	2	2	118	132	283	3.76	1789	17	0.95	0.48
9471T	4	5	4	1	0	2	2	0	110	128	281	3.65	1788	21	1.17	0.63
9515T	6	2	7	0	0	4	3	2	120	144	284	4.10	1670	18	1.08	0.48
9567T	1	0	4	0	0	3	0	0	64	72	134	2.05	1815	16	0.88	0.14
MEAN	9	4	7	0	0	3	3	1	148	174	370	5	1694	35	2	1
SD	27	8	8	1	1	3	3	1	186	231	458	7	161	70	4	2
MIN	0	0	1	0	0	0	0	0	28	32	82	1	1259	13	1	0
MAX	157	43	51	2	3	15	15	5	1224	1508	2932	43	1945	441	23	12
MEDIAN	3	2	6	0	0	3	2	0	112	128	281	4	1730	21	1	0

Footnotes:

*Effect was predicted using the Variant Effect Predictor (VEP) v108

**Includes HIGH, MODERATE, LOW, and MODIFIER classifications

***TMB=tumor mutation burden

****MSI=microsatellite instability detected using MSIsensor2 v0.1

*****MMR-D was calculated based on the cut-off set to 1.5 indels in homopolymer regions per Mb according to Albayrak et al. 2020 (doi: 10.1200/PO.20.00185)

Supplemental Table S3: TOP 20 most frequently altered genes in mCLM samples

Hugo_Symbol	Frame_Shift_Del	Frame_Shift_Ins	In_Frame_Del	In_Frame_Ins	Missense_Mutation	Nonsense_Mutation	Nonstop_Mutation	Splice_Site	Translation_Start_Site	total	AlteredSamples
TP53	2	2	0	0	19	8	0	3	0	34	31
APC	11	2	0	0	3	26	0	0	0	42	27
KRAS	0	0	0	0	17	0	0	0	0	17	17
ABCA13	0	0	0	0	9	1	0	0	0	10	8
PIK3CA	0	0	0	0	10	0	0	0	0	10	8
PCLO	0	0	0	0	9	0	0	0	0	9	8
UNC80	0	0	0	0	9	0	0	0	0	9	8
FAT4	0	0	0	0	8	0	0	0	0	8	8
COL6A5	1	0	0	0	6	0	0	0	0	7	7
HRNR	0	0	0	0	6	1	0	0	0	7	7
ZFHX4	0	0	0	0	8	1	0	0	0	9	6
RYR1	0	0	0	0	8	0	0	0	0	8	6
FBXW7	0	0	0	0	6	1	0	0	0	7	6
RHPN2	0	0	0	0	2	5	0	0	0	7	6
VIPR2	1	0	0	0	6	0	0	0	0	7	6
ADAMTS16	0	0	0	0	6	0	0	0	0	6	6
CCDC168	1	1	0	0	4	0	0	0	0	6	6
FNDC1	0	0	0	0	6	0	0	0	0	6	6
MUC12	0	0	0	0	5	1	0	0	0	6	6
RYR2	0	0	0	0	6	0	0	0	0	6	6

FLAGs (*TTN, AHNAK2, DNAH17, SYNE1, MUC16* , and *OBSCN*) according to Shyr et al. 2014 (doi: 10.1186/s12920-014-0064-y) excluded from the list

Supplemental Table S4: Basic description of somatic copy number variations in mCLM

Patient ID	Tumor purity	CNVs total	CNV_0	CNV_1	CNV_2	CNV_3	CNV_>3	Average_size (bp)
6315T	0.73	71	3	39	0	23	6	16387100
6763T	0.55	57	0	24	0	23	10	12400900
6783T	0.72	53	2	27	0	19	5	18160500
6807T	0.95	151	3	41	0	35	72	9224750
6816T	0.87	72	1	30	0	32	9	13601900
6824T	0.75	81	2	21	0	34	24	13134500
6835T	0.88	52	1	19	0	23	9	11862800
6856T	0.79	56	1	32	0	17	6	22714900
6869T	0.15	19	1	12	0	4	2	8241010
6999T	0.69	129	4	40	0	60	25	10798700
7006T	0.29	16	1	8	0	6	1	9128010
7011T	0.87	66	1	21	0	28	16	9971290
7047T	0.95	46	2	27	0	7	10	14330200
7081T	0.82	62	0	31	0	28	3	12500700
7084T	0.76	57	0	5	0	37	15	7502370
7247T	0.52	64	0	27	0	34	3	12404800
7295T	0.29	20	0	15	0	5	0	7694900
7322T	0.72	46	0	14	0	25	7	5992710
7960T	0.15	24	0	3	0	16	5	6455150
7968T	0.79	19	0	6	0	13	0	7709880
7976T	0.74	82	5	29	0	36	12	17424700
8008T	0.69	48	1	20	0	19	8	9935350
8040T	0.94	20	0	12	0	8	0	18704700
8048T	0.76	99	3	42	0	32	22	11044800
8164T	0.32	22	3	16	0	3	0	7060940
8169T	0.77	115	3	32	0	59	21	11231400
8174T	0.72	58	0	36	0	21	1	27608500
8689T	0.84	50	0	6	0	27	17	3208230
8837T	0.77	30	0	4	0	20	6	8179590
8928T	0.95	64	4	38	0	14	8	13532300
8943T	0.81	45	0	4	0	30	11	6185390
9181T	0.75	20	0	6	0	12	2	9084810
9224T	0.76	33	0	16	0	17	0	19325400
9252T	0.75	33	1	7	0	18	7	4674240
9256T	0.9	80	1	41	0	27	11	15953800
9277T	0.65	57	0	15	0	30	12	11987300
9285T	0.78	71	1	20	0	31	19	9854020
9455T	0.81	34	0	3	0	27	4	5451150
9471T	0.62	87	4	57	0	25	1	11504441
9515T	0.75	40	2	15	0	23	0	10693400
9567T	0.72	42	0	33	0	9	0	15450400
MEAN	0.708	56	1	22	0	23	10	11666145
S.D.	0.223	28	1	14	0	12	11	5185252
MIN	0.150	16	0	3	0	3	0	3208230
MAX	0.950	151	5	57	0	60	72	27608500
MEDIAN	0.750	53	1	20	0	23	7	11044800

Abbreviations: CNV = copy number variation

CNV_0 = CNV with zero copies

CNV_1 = CNVs with one copy

CNV_2 = CNVs with two copies

CNV_3 = CNVs with three copies

CNV_>3 = CNVs with more than three copies

Supplemental Table S5: Relative contributions all 79 reference SBS mutational signatures in COSMIC v3.3 (see Materials and Methods)

sample	SBS1	SBS2	SBS3	SBS4	SBS5	SBS6	SBS7a	SBS7b	SBS7c	SBS7d	SBS8	SBS9	SBS10a	SBS10b	SBS10c	SBS10d	SBS11	SBS12	SBS13	SBS14
6315T	0.199	0.057	0	0	0	0.159	0	0	0	0	0	0	0	0	0	0	0	0	0	0
6763T	0.26	0	0	0	0	0	0	0	0	0	0	0	0	0	0	0	0	0	0	0
6783T	0.241	0	0	0	0	0.055	0	0	0	0	0	0	0	0	0	0	0	0	0	0
6807T	0	0	0	0	0	0.118	0	0	0	0	0	0	0	0	0	0	0	0	0	0
6816T	0	0	0	0	0	0	0	0	0	0	0	0	0	0	0	0	0	0	0	0
6824T	0.174	0	0	0	0	0.185	0	0	0	0	0	0	0	0.0565	0	0	0	0	0	0
6835T	0.29	0	0	0	0	0	0	0	0	0	0	0	0	0	0	0	0	0	0	0
6856T	0.283	0	0	0	0	0	0	0	0	0	0	0	0	0	0	0	0	0	0	0
6869T	0.166	0.065	0	0	0	0	0	0	0	0	0	0	0	0	0	0	0	0	0	0
6999T	0.055	0	0	0	0	0.201	0	0	0	0	0.105	0	0	0	0	0	0	0	0.059	0
7006T	0.117	0	0	0	0	0	0	0	0.098	0	0	0	0	0	0	0	0	0	0	0
7011T	0.213	0	0	0	0	0	0	0.053	0	0	0	0	0	0	0	0	0	0	0	0
7047T	0.376	0	0	0.095	0	0	0	0	0	0	0	0	0	0.0599	0	0	0	0	0	0
7081T	0.095	0	0	0	0	0.088	0	0	0	0	0	0	0	0.0582	0	0	0	0	0	0
7084T	0.138	0	0	0	0	0.087	0	0	0	0	0	0	0	0	0	0	0	0	0	0
7247T	0.177	0	0	0	0	0	0	0.063	0	0	0	0	0	0	0	0	0	0	0	0
7295T	0	0	0	0	0	0.112	0	0	0	0	0	0	0	0	0	0	0	0	0	0
7322T	0.197	0	0	0	0	0	0	0	0	0	0	0	0	0	0	0	0	0	0	0
7960T	0	0	0	0	0	0	0	0	0	0	0	0	0	0	0	0	0	0.065	0	0
7968T	0.171	0	0	0	0	0	0	0.091	0	0	0	0	0	0	0	0	0	0	0	0
7976T	0.202	0	0	0	0	0	0	0	0	0	0.054	0	0	0	0	0	0	0	0	0
8008T	0	0	0	0.096	0	0.097	0	0	0	0	0	0	0	0	0	0	0	0	0	0
8040T	0.287	0	0	0	0	0	0	0	0	0	0	0	0	0	0	0	0	0	0	0
8048T	0.09	0	0.157	0	0	0.09	0	0	0	0	0.19	0	0	0	0	0	0	0	0	0
8164T	0.385	0	0	0	0	0	0	0	0	0	0	0	0	0	0	0	0	0	0	0
8169T	0.115	0	0	0	0	0.071	0	0	0	0	0	0	0	0	0	0	0	0	0	0
8174T	0.231	0	0	0	0	0.112	0	0	0	0	0	0	0	0	0	0	0	0	0.057	0
8689T	0.195	0	0	0.186	0	0.078	0.061	0	0	0	0	0	0	0	0	0	0	0	0	0
8837T	0	0	0	0	0	0.251	0	0	0	0	0	0	0	0	0	0	0	0.122	0	0
8928T	0.072	0	0	0	0	0	0	0	0	0	0	0	0	0	0	0	0	0	0	0
8943T	0.175	0	0	0	0	0.149	0	0	0	0	0	0	0	0	0	0	0	0	0	0
9181T	0.249	0	0	0	0	0.12	0	0	0	0	0	0	0	0	0	0	0	0	0	0
9224T	0.174	0	0	0	0	0	0	0	0	0	0.109	0	0	0	0	0	0	0	0	0
9252T	0.06	0	0	0	0	0.184	0	0	0	0	0	0	0	0	0	0	0	0	0	0
9256T	0.21	0	0	0	0	0	0	0	0	0	0	0	0	0	0	0	0	0	0	0
9277T	0.058	0	0	0	0	0.129	0	0.06	0	0	0	0	0	0	0	0	0	0	0.054	0
9285T	0.169	0	0	0	0	0.19	0	0	0	0	0	0	0	0	0	0	0	0	0	0
9455T	0.181	0	0	0	0	0	0	0	0	0	0	0	0	0	0	0	0	0	0	0
9471T	0	0	0	0	0	0.06	0	0	0	0	0	0	0	0	0	0	0	0	0	0
9515T	0.127	0	0	0	0	0	0	0	0	0	0	0	0	0	0	0	0	0	0	0
9567T	0.144	0	0	0	0	0.174	0	0.089	0	0	0	0	0	0	0	0	0	0	0	0

sample	SBS15	SBS16	SBS17a	SBS17b	SBS18	SBS19	SBS20	SBS21	SBS22	SBS23	SBS24	SBS25	SBS26	SBS27	SBS28	SBS29	SBS30	SBS31	SBS32
6315T	0.07	0	0	0	0	0	0	0	0	0	0	0	0	0	0	0	0	0	0
6763T	0.305	0	0	0	0	0	0	0	0	0	0	0	0	0	0	0	0	0	0
6783T	0.12	0	0	0	0	0	0	0	0	0.054	0	0	0	0	0	0	0	0	0
6807T	0.182	0	0	0	0	0	0	0	0	0	0	0	0	0	0	0	0	0	0
6816T	0.163	0	0	0.1112	0	0	0	0	0.051	0	0	0	0	0	0	0	0	0	0
6824T	0.172	0	0	0	0.092	0	0	0	0	0	0	0	0	0	0	0	0	0	0
6835T	0.257	0	0	0	0	0	0	0	0	0	0.086	0	0	0	0	0	0	0	0
6856T	0.143	0.073	0	0	0	0	0	0	0	0	0	0	0	0	0	0	0	0.091	0.082
6869T	0.218	0	0	0	0	0	0.062	0	0	0	0	0	0	0	0	0	0	0	0
6999T	0.103	0	0	0	0	0	0	0	0	0	0	0	0	0	0	0.071	0	0	0
7006T	0.173	0	0	0	0	0	0	0	0.128	0	0	0	0	0	0	0	0	0	0
7011T	0.253	0	0	0	0	0	0	0	0	0	0	0	0	0	0	0	0	0	0
7047T	0.232	0	0	0	0	0	0	0	0	0	0	0	0	0	0	0	0	0	0
7081T	0.162	0	0	0	0	0	0	0	0	0	0	0	0	0	0	0	0	0	0
7084T	0.092	0	0	0.091	0	0	0	0	0	0	0	0	0	0	0	0	0	0	0
7247T	0.296	0	0	0	0	0	0	0	0	0	0	0	0	0	0	0.081	0	0	0
7295T	0.097	0	0	0.0733	0	0	0	0	0	0	0.053	0	0	0	0	0	0	0	0
7322T	0.373	0	0	0	0	0	0	0	0	0	0	0	0	0	0	0	0	0	0
7960T	0.333	0	0	0.1333	0	0	0	0	0	0	0	0	0	0	0	0	0	0	0
7968T	0.225	0	0	0	0	0	0	0	0	0	0.069	0	0	0	0	0	0	0.055	0
7976T	0.211	0	0	0	0	0	0	0	0	0	0.08	0	0	0	0	0	0	0.128	0
8008T	0.098	0	0	0.206	0	0	0	0	0	0	0	0	0	0	0	0	0	0	0
8040T	0.302	0	0	0	0	0	0	0	0	0	0	0	0	0	0	0.111	0	0	0
8048T	0.096	0	0	0	0	0	0	0	0	0	0	0	0	0	0	0	0	0	0
8164T	0.46	0	0	0	0	0	0	0	0	0	0	0	0	0	0	0	0	0	0
8169T	0.173	0	0	0.1966	0	0	0	0	0	0	0	0	0	0	0	0	0	0	0
8174T	0.072	0	0	0	0	0	0	0	0	0	0	0	0	0	0	0	0	0	0
8689T	0	0	0	0	0	0	0	0	0	0	0	0	0	0	0	0	0	0	0
8837T	0	0.058	0	0	0.093	0	0	0	0	0	0	0	0	0	0	0	0.093	0	0
8928T	0.349	0	0	0	0	0	0	0	0.056	0	0	0	0	0	0	0	0	0.058	0
8943T	0.107	0	0	0	0.072	0	0	0	0	0	0	0	0	0	0	0	0	0	0
9181T	0.113	0	0	0	0	0	0	0	0	0	0	0	0	0	0	0	0	0	0
9224T	0.218	0	0	0.0801	0	0	0	0	0	0	0	0	0	0	0	0	0	0	0
9252T	0.375	0	0	0	0	0	0	0	0	0	0	0	0	0	0	0	0	0	0
9256T	0.265	0	0	0	0	0	0	0	0	0	0	0	0	0	0	0.053	0	0	0
9277T	0.052	0	0	0	0	0	0	0	0.064	0	0.071	0	0	0	0	0	0	0	0
9285T	0.106	0	0	0	0.059	0	0	0	0	0	0	0	0	0	0	0	0	0	0
9455T	0.216	0	0	0	0.052	0	0	0	0	0	0	0	0	0	0	0	0	0	0
9471T	0.18	0	0	0	0	0	0	0	0	0	0.132	0	0	0	0	0	0	0	0
9515T	0.292	0.06	0	0	0	0	0	0	0	0	0	0	0	0	0	0	0	0.057	0
9567T	0.262	0	0	0	0	0	0	0	0	0	0	0	0	0	0	0	0	0	0

sample	SBS33	SBS34	SBS35	SBS36	SBS37	SBS38	SBS39	SBS40	SBS41	SBS42	SBS43	SBS44	SBS45	SBS46	SBS47	SBS48	SBS49	SBS50	SBS51	SBS52	SBS53
6315T	0	0	0	0	0	0	0	0	0	0	0	0	0	0	0	0	0	0	0	0	0
6763T	0	0	0	0	0	0	0	0	0	0	0	0	0	0	0	0	0	0	0	0	0
6783T	0	0	0	0	0	0	0	0	0	0	0	0	0	0	0	0	0	0	0	0	0
6807T	0	0	0	0	0	0	0	0	0	0	0	0	0	0	0	0	0	0	0	0	0
6816T	0	0	0	0	0	0	0	0	0	0	0	0	0	0.156	0	0	0	0	0	0	0
6824T	0	0	0	0	0	0	0	0	0	0	0	0	0	0	0	0	0	0	0	0	0
6835T	0	0	0	0	0	0	0	0	0	0	0	0	0	0.058	0	0	0	0	0.081	0	0
6856T	0	0	0	0	0	0	0	0	0	0	0	0	0	0	0	0	0	0.062	0	0	0
6869T	0	0	0.121	0	0	0	0	0	0	0	0	0	0	0	0	0	0	0	0	0	0
6999T	0	0	0	0	0	0	0.091	0	0	0	0	0	0	0	0	0	0	0	0	0	0
7006T	0	0	0	0	0	0	0	0	0	0.129	0	0	0	0	0	0	0	0	0	0	0
7011T	0	0	0	0	0	0	0	0	0	0	0	0	0	0.065	0	0	0	0	0	0	0
7047T	0	0	0	0	0	0	0	0	0	0	0	0	0	0	0	0	0	0	0	0	0
7081T	0	0	0	0	0	0	0	0	0	0	0	0	0	0	0	0	0	0	0	0	0
7084T	0	0	0	0	0	0	0	0	0	0	0	0	0	0	0	0	0	0	0	0	0
7247T	0	0	0	0	0	0	0	0	0	0	0	0	0	0	0	0	0	0	0	0	0
7295T	0	0	0	0	0	0	0	0	0	0.078	0	0	0	0	0	0	0	0	0	0	0
7322T	0	0	0	0	0	0	0	0	0	0	0	0	0	0	0	0	0	0	0	0	0
7960T	0	0	0	0	0	0	0	0	0	0	0.055	0	0	0.066	0	0	0	0	0	0	0
7968T	0	0	0	0	0	0	0	0	0	0	0	0	0	0	0	0	0	0	0	0	0
7976T	0	0	0	0	0	0	0	0	0	0	0	0	0	0	0	0	0	0	0	0	0
8008T	0	0	0	0	0	0	0	0	0	0	0	0	0	0	0	0	0	0	0	0	0
8040T	0	0	0	0	0	0	0	0	0	0	0	0	0	0	0	0	0	0	0	0	0
8048T	0	0	0	0	0	0	0.186	0	0	0	0	0	0	0	0	0	0	0	0	0	0
8164T	0	0	0	0	0	0	0	0	0	0	0	0	0	0	0	0	0	0	0	0	0
8169T	0	0	0	0	0	0	0	0	0	0	0	0	0	0	0	0	0	0	0	0	0
8174T	0	0	0.061	0	0	0	0	0	0	0.056	0	0	0	0	0	0	0	0	0	0	0
8689T	0	0	0	0	0	0	0	0	0	0.053	0	0	0	0	0	0	0	0	0	0	0
8837T	0	0	0	0	0	0	0	0	0	0	0	0.104	0	0	0	0	0	0	0	0	0
8928T	0	0	0	0	0	0	0	0	0	0	0	0	0	0	0	0	0	0	0	0	0
8943T	0	0	0	0	0	0	0	0	0	0	0	0	0	0.114	0	0	0	0	0	0	0
9181T	0	0	0	0	0	0	0	0	0	0	0	0	0	0	0	0	0	0	0	0	0
9224T	0	0	0	0	0	0	0	0	0	0	0	0	0	0	0	0	0	0	0	0	0
9252T	0	0	0	0	0	0	0	0	0	0	0	0	0	0	0	0	0	0	0	0.056	0
9256T	0	0	0	0	0	0	0	0	0	0	0	0	0	0	0	0	0	0	0	0	0
9277T	0	0	0	0	0	0	0.079	0	0	0	0	0	0	0	0	0	0	0	0	0	0
9285T	0	0	0	0	0	0	0	0	0	0	0	0	0	0	0	0	0	0	0	0	0
9455T	0	0	0	0	0	0	0	0	0	0	0	0	0	0	0	0	0	0	0	0	0
9471T	0	0	0	0	0	0	0	0	0	0	0.052	0	0	0	0	0	0	0	0	0	0
9515T	0	0	0	0	0	0	0	0	0	0	0	0	0	0	0	0	0	0	0	0	0
9567T	0	0	0	0	0	0	0	0	0	0	0	0	0	0	0	0	0	0	0	0	0

sample	SBS54	SBS55	SBS56	SBS57	SBS58	SBS59	SBS60	SBS84	SBS85	SBS86	SBS87	SBS88	SBS89	SBS90	SBS91	SBS92	SBS93	SBS94
6315T	0	0	0	0	0.083	0	0	0	0	0	0.192	0	0	0	0	0	0	0
6763T	0	0	0	0	0	0	0	0	0	0	0.076	0.059	0	0	0	0	0	0
6783T	0	0	0	0	0	0	0	0	0	0.066	0.175	0	0	0	0	0	0	0
6807T	0.051	0	0	0	0	0	0	0	0	0	0.211	0	0	0	0	0	0	0
6816T	0	0	0	0	0	0	0	0	0	0	0.168	0	0	0	0	0	0	0
6824T	0	0	0	0	0	0	0	0	0	0	0.252	0	0	0	0	0	0	0
6835T	0	0	0	0	0	0	0	0	0	0	0	0	0	0	0	0	0	0
6856T	0	0	0	0	0	0	0	0	0	0.094	0	0	0	0	0	0	0	0
6869T	0.073	0	0	0	0	0	0	0	0	0	0.15	0	0	0	0	0	0	0
6999T	0	0	0	0	0	0	0	0	0	0	0.085	0	0	0	0	0	0	0
7006T	0	0	0	0	0.056	0	0	0	0	0	0.074	0	0	0	0	0	0	0
7011T	0	0	0	0	0	0	0	0	0	0	0	0	0	0	0	0	0	0
7047T	0	0	0	0	0	0	0	0	0	0	0	0	0	0	0	0	0	0
7081T	0	0	0	0	0	0	0	0	0	0	0.224	0	0	0	0	0	0	0
7084T	0	0	0	0	0	0	0	0	0	0	0.133	0	0	0	0	0	0	0.078
7247T	0	0	0	0	0	0	0	0	0	0	0.175	0	0	0	0	0	0	0
7295T	0.087	0	0	0	0	0	0	0	0	0	0.207	0	0	0	0	0	0	0
7322T	0	0	0	0	0	0	0	0	0	0	0.219	0	0	0	0	0	0	0
7960T	0.205	0	0	0	0	0	0	0	0	0	0	0	0	0	0	0	0	0
7968T	0	0	0	0	0	0	0	0	0	0	0.192	0	0	0	0	0	0	0
7976T	0	0	0	0	0	0	0	0	0	0	0	0	0	0	0	0	0	0
8008T	0	0	0	0	0.059	0	0	0	0	0	0.132	0	0	0	0	0	0	0
8040T	0	0	0	0	0	0	0	0	0	0	0.135	0	0	0	0	0	0	0
8048T	0	0	0	0	0	0	0	0	0	0	0	0	0	0	0	0	0	0
8164T	0	0	0	0	0	0	0	0	0	0	0.105	0	0	0	0	0	0	0
8169T	0	0	0	0	0	0	0	0	0	0	0.17	0	0	0	0	0	0	0
8174T	0	0	0	0	0	0	0	0	0	0	0.177	0	0	0	0	0	0	0
8689T	0.05	0	0	0	0	0	0	0	0	0	0.183	0	0	0	0	0	0	0
8837T	0	0	0	0	0	0	0	0	0	0	0.064	0	0	0	0	0	0	0
8928T	0	0	0	0	0	0	0	0	0	0	0.232	0	0	0	0	0	0	0
8943T	0	0	0	0	0	0	0	0	0	0	0.253	0	0	0	0	0	0	0
9181T	0	0	0	0	0	0	0	0	0	0	0.313	0	0	0	0	0	0	0
9224T	0	0	0	0	0	0	0	0	0	0	0.141	0	0	0	0	0	0	0
9252T	0	0	0	0	0	0	0	0	0	0	0.124	0	0	0	0	0	0	0
9256T	0	0	0	0	0	0	0	0	0	0	0.19	0	0	0	0	0	0	0
9277T	0	0	0	0	0	0	0	0	0	0	0.109	0	0	0	0	0	0.052	0
9285T	0	0	0	0	0	0	0	0	0	0	0.244	0	0	0	0	0	0	0
9455T	0	0	0	0	0	0	0	0	0	0	0.126	0	0	0	0	0	0	0
9471T	0	0	0	0	0	0	0	0	0	0.055	0.115	0	0	0	0	0	0	0
9515T	0.056	0	0	0	0	0	0	0	0	0	0.182	0	0	0	0	0	0	0
9567T	0	0	0	0	0	0	0	0	0	0	0.236	0	0	0	0	0	0	0

Supplementary Table S6: Relative contributions of the top 9 most significant reference SBS mutation signatures (COSMIC v3.3, see Materials and Methods)

**sampleID**	**SBS1**	**SBS6**	**SBS7b**	**SBS15**	**SBS17b**	**SBS18**	**SBS24**	**SBS31**	**SBS87**
6315T	0.158885	0.126631	0.100324	0.134739	0	0.076445	0.068594	0	0.305909
6763T	0.211256	0	0	0.357085	0	0	0.111301	0.079537	0.189878
6783T	0.192696	0	0	0.180233	0	0.085302	0.050319	0.115034	0.280149
6807T	0	0.07676	0	0.244452	0	0.085316	0.096639	0.101594	0.328646
6816T	0	0	0	0.228366	0.125401	0.064538	0.145518	0.129894	0.306283
6824T	0.171018	0.200383	0	0.169579	0	0.139984	0	0	0.279508
6835T	0.248003	0	0	0.293852	0	0.073539	0.13221	0.059083	0.117387
6856T	0.258167	0	0.080299	0.180072	0	0.103259	0.096365	0.153084	0.102147
6869T	0.108453	0	0.068267	0.282895	0	0.07618	0.123678	0.073808	0.263989
6999T	0	0.102373	0.053071	0.201489	0	0.09845	0.164344	0.074775	0.263445
7006T	0	0	0	0.291269	0	0	0.15575	0.185676	0.314717
7011T	0.165962	0	0.076741	0.297382	0	0.18039	0.06271	0.059102	0.134823
7047T	0.345481	0	0	0.261112	0	0.123307	0	0	0.112355
7081T	0.052646	0.054825	0	0.231403	0	0.093951	0	0.098452	0.376937
7084T	0.096699	0	0.071265	0.181021	0.104455	0.135844	0.076221	0	0.285735
7247T	0.163452	0	0.060842	0.313732	0	0.09629	0.075591	0	0.238725
7295T	0	0.13265	0	0.142604	0.088436	0.087403	0.123795	0.072156	0.31337
7322T	0.16442	0	0	0.403044	0	0.091675	0	0	0.290909
7960T	0	0	0	0.361942	0.157842	0	0.154261	0.168753	0.131227
7968T	0.156674	0	0.106145	0.253814	0	0	0.106826	0.070597	0.249643
7976T	0.170018	0	0	0.236969	0	0.136731	0.137143	0.189705	0.114589
8008T	0	0	0	0.164591	0.219646	0.097498	0.110584	0.1121	0.237479
8040T	0.272164	0	0	0.316258	0	0.1019	0.066961	0	0.18243
8048T	0	0	0	0.213317	0	0.086995	0.178899	0.212454	0.232246
8164T	0.385483	0	0	0.460022	0	0	0	0	0.104603
8169T	0.086705	0	0	0.22654	0.205114	0.074585	0.053721	0	0.258704
8174T	0.178101	0.10966	0.062351	0.128713	0	0.112483	0.079823	0	0.296179
8689T	0.137814	0.077346	0.10937	0	0	0.129379	0.176512	0	0.321373
8837T	0	0.334188	0	0	0	0.214881	0.147762	0.140307	0.13743
8928T	0	0	0	0.384482	0	0.113545	0	0.11497	0.328588
8943T	0.138907	0.161451	0	0.136012	0	0.131514	0	0	0.338333
9181T	0.238096	0.085187	0	0.151456	0	0	0.063587	0	0.383257
9224T	0.130092	0	0	0.255493	0.089489	0.065518	0.090763	0.118481	0.250165
9252T	0	0.100125	0.055689	0.462899	0	0	0.09713	0	0.248604
9256T	0.182609	0	0.076409	0.311653	0	0	0.088817	0	0.282356
9277T	0	0.054135	0.066504	0.157722	0	0.133814	0.13721	0.090423	0.317052
9285T	0.155724	0.183953	0.067395	0.126375	0	0.139024	0	0	0.29606
9455T	0.122384	0.050556	0	0.253618	0	0.120623	0.066935	0.088217	0.240188
9471T	0	0	0	0.23755	0	0.109126	0.222095	0.153825	0.232241
9515T	0.093017	0	0	0.321478	0	0.063605	0.098917	0.104524	0.285232
9567T	0.139365	0.173725	0.097359	0.266192	0	0.065744	0	0	0.251232

Cosine similarities (see e.g. Blokzijl et al. 2018*) of reconstructed mutational catalogues to the mutational profile of original sample
when fitting only the top 9 most significant reference signatures vs the full COSMIC v3.3 reference signature set (n=79)

*Blokzijl F et al. Genome Med. 2018 (doi: 10.1186/s13073-018-0539-0)

Supplemental Table S7: TOP 20 most frequently altered genes in non-malignant liver samples

	CTU2	*GGT3P*	*AGAP6*	*KMT2C*	*SVEP1*	*ZNF101*	*CCDC7*	*MYH7B*	*PRAMEF10*	*CDH23*	*GAA*	*TXNDC2*	*SPTBN5*	*CATSPER2*	*CCDC136*	*NEB*	*AGL*	*ATP13A5*	*DHRS4L2*	*KMT2E*
6315Z	1	0	0	0	1	0	0	0	0	0	1	0	0	0	1	0	0	0	1	0
6763Z	1	0	0	0	0	0	0	0	0	0	1	0	0	0	0	0	0	0	0	0
6783Z	1	0	0	0	0	1	0	0	0	0	0	0	0	0	0	0	1	0	0	0
6807Z	1	1	1	0	0	1	1	0	0	1	0	1	0	0	0	0	0	1	0	0
6816Z	1	1	1	1	0	0	1	0	0	0	0	0	0	1	0	0	0	0	0	1
6824Z	1	0	0	0	1	0	0	0	1	0	1	1	0	0	0	0	0	0	0	0
6835Z	1	1	0	0	0	0	0	1	0	0	0	0	0	1	0	0	0	0	0	0
6856Z	1	0	0	1	1	0	0	1	1	1	1	1	0	0	0	0	0	0	1	1
6869Z	1	0	0	0	1	0	0	0	0	0	0	0	0	1	0	1	0	0	0	0
6999Z	1	1	0	0	1	1	0	1	0	1	0	1	1	1	0	0	1	0	0	0
7006Z	1	1	1	0	0	0	0	0	0	0	0	0	0	1	0	0	0	1	0	0
7011Z	1	0	1	1	0	0	0	0	1	1	1	1	0	0	1	0	0	0	0	0
7047Z	1	1	0	1	0	1	0	1	1	0	0	0	0	0	1	1	0	0	0	0
7081Z	1	1	0	0	0	0	0	1	1	0	1	0	0	0	0	0	1	0	0	0
7084Z	1	0	0	0	1	0	0	0	0	0	1	0	0	0	0	0	1	0	1	0
7247Z	1	1	1	1	0	1	0	0	0	0	0	0	0	0	1	0	0	0	0	0
7295Z	0	1	0	1	0	0	0	0	1	0	0	1	1	1	0	0	0	0	0	0
7322Z	1	0	0	1	0	0	0	0	0	0	0	0	1	0	0	0	0	0	0	1
7960Z	1	0	1	1	0	0	0	1	0	0	0	0	0	0	0	0	0	0	0	0
7968Z	1	1	1	0	0	0	0	0	0	0	1	0	0	0	0	1	1	0	0	0
7976Z	1	0	0	0	0	1	0	0	0	1	0	0	0	0	0	0	0	0	0	0
8008Z	1	1	0	0	1	0	0	0	1	0	0	0	0	1	0	0	0	0	1	1
8040Z	1	0	1	0	0	1	0	0	1	1	0	0	1	0	0	0	0	1	0	1
8048Z	1	0	0	1	1	1	0	0	0	1	0	0	0	0	0	0	0	0	0	0
8164Z	1	0	0	0	0	1	1	1	0	0	0	0	0	0	0	0	1	0	1	1
8169Z	1	1	0	0	0	0	0	1	0	0	0	1	1	0	1	0	0	0	0	0
8174Z	1	1	0	0	0	0	1	0	0	0	0	0	0	0	0	1	0	0	0	0
8689Z	1	0	1	1	0	0	1	1	0	0	0	1	0	0	0	1	0	0	0	0
8837Z	1	0	0	0	1	0	1	0	0	0	1	0	0	0	0	0	0	0	0	0
8928Z	1	0	0	0	0	1	0	0	0	1	0	1	0	0	1	0	0	1	0	0
8943Z	1	0	1	0	1	0	1	1	1	0	0	0	0	0	1	0	1	0	1	0
9181Z	1	1	1	0	0	0	0	0	1	0	1	0	0	0	1	0	0	1	0	0
9224Z	1	0	1	1	0	0	1	0	0	0	0	1	1	0	0	0	0	0	0	0
9252Z	1	1	0	0	0	0	0	1	0	1	0	0	0	0	1	0	0	1	1	1
9256Z	1	1	0	0	0	0	0	0	0	0	0	0	1	0	0	1	0	1	1	0
9277Z	1	1	0	0	1	0	1	0	0	0	0	0	0	1	0	1	0	1	0	0
9285Z	1	0	0	0	1	0	1	0	1	0	0	0	1	0	0	1	0	0	0	0
9455Z	1	1	1	1	0	1	0	0	0	0	0	1	1	0	1	1	1	0	0	0
9471Z	1	0	1	1	0	0	0	0	0	0	0	0	1	1	0	0	0	0	0	1
9515Z	1	1	0	0	0	0	1	0	1	1	1	0	0	1	0	0	0	0	1	0
9567Z	0	0	0	0	1	1	1	1	0	1	0	0	0	0	0	0	1	1	0	1
Total	39	19	14	13	13	12	12	12	12	11	11	11	10	10	10	9	9	9	9	9

Numbers of variants with HIGH or MODERATE effect for each patient displayed

Supplemental Table S8: Functionally relevant list of somatic and gemline variants in HRR and MMR genes in mCLM

Patient ID	HRR genes somatic	HRR genes germline	MMR genes somatic	MMR genes germline
6315T				
6763T				
6783T				
6807T				
6816T				
6824T				
6835T				*MLH1* -p.V15A
6856T				
6869T				
6999T	*BRCA2* -p.V1532Lfs*11			
7006T				
7011T				
7047T	*NBN* -p.R466Kfs*5			
7081T				
7084T				
7247T				
7295T				
7322T				
7960T			*MLH3* -p.T618Nfs*2 & *MSH3* -p.K714Rfs*24 & TMB-H/MSI-H	
7968T	*BRCA1* -p.K748*			
7976T				
8008T	*ATR* -p.Q414delinsH*			
8040T				*MLH1* -p.K618E
8048T	*BRCA1* -p.N593Ifs*6	*BRCA2* -p.G1529R		
8164T			TMB-H/MSI-H	
8169T				
8174T				
8689T				
8837T				
8928T				
8943T		*RAD51C* -p.T287A		
9181T			*MSH3* -p.Q852*	
9224T		*ATM* -p.A2274T		
9252T				
9256T				
9277T				
9285T		*ATM* -p.X946_splice		
9455T				
9471T				
9515T				
9567T				

Only variants classified as HIGH in terms of functional impact predictions are listed

HRR genes followed (Norquist et al. 2018, doi: 10.1158/1078-0432.CCR-17-1327)

ATM
ATR
BARD1
BLM
BRCA1
BRCA2
BRIP1
CHEK2
MRE11A
NBN
PALB2
RAD51C
RAD51D
RBBP8
SLX4
XRCC2

MMR genes followed (Shirts et al. 2018, doi: 10.1016/j.ajhg.2018.05.001)

MLH1
MLH3
MSH2
MSH3
MSH6
PMS2

Supplemental Table S9: List of genes used for evaluation of somatic variants in oncodriver pathways in mCLM

For pathway analysis, gene lists from Sanchez-Vega et al. 2018 (doi: 10.1016/j.cell.2018.03.035) and Kim & Bodmer 2022 (doi: 10.1007/s00432-021-03888-w),
supplemented by pathway definitions in KEGGs GSEA database v7.5.1

Pathways - genes used in combination for each pathway analysis, N=13

**Cell_Cycle**	**Hippo**	**MYC**	**NOTCH**	**NRF2**	**PI3K**	**TGF-Beta**	**RTK-RAS**	**TP53**	**WNT**	**Hedgehog**	**TRAIL**	**JAKSTAT**
CDKN1A	*STK4*	*MAX*	*ARRDC1*	*NFE2L2*	*EIF4EBP1*	*TGFBR1*	*ABL1*	*TP53*	*CHD8*	*CSNK1A1L*	*TNFRSF10A*	*AKT3*
CDKN1B	*STK3*	*MGA*	*CNTN6*	*KEAP1*	*AKT1*	*TGFBR2*	*EGFR*	*MDM2*	*LEF1*	*CSNK1A1*	*PIK3CD*	*SPRY3*
CDKN2A	*SAV1*	*MLX*	*CREBBP*	*CUL3*	*AKT2*	*ACVR2A*	*ERBB2*	*MDM4*	*LGR4*	*CSNK1D*	*PIK3R2*	*SPRY1*
CDKN2B	*LATS1*	*MLXIP*	*EP300*		*AKT3*	*ACVR1B*	*ERBB3*	*ATM*	*LGR5*	*CSNK1E*	*TNFRSF10B*	*SPRY2*
CDKN2C	*LATS2*	*MLXIPL*	*HES1*		*AKT1S1*	*SMAD2*	*ERBB4*	*CHEK2*	*LRP5*	*CSNK1G2*	*TNFRSF10C*	*STAM2*
CCND1	*MOB1A*	*MNT*	*HES2*		*DEPDC5*	*SMAD3*	*PDGFRA*	*RPS6KA3*	*LRP6*	*CSNK1G3*	*IKBKB*	*IRF9*
CCND2	*MOB1B*	*MXD1*	*HES3*		*DEPTOR*	*SMAD4*	*PDGFRB*		*LZTR1*	*FBXW11*	*CHUK*	*PIAS3*
CCND3	*YAP1*	*MXD3*	*HES4*		*INPP4B*		*MET*		*NDP*	*GAS1*	*CFLAR*	*IL24*
CCNE1	*WWTR1*	*MXD4*	*HES5*		*MAPKAP1*		*FGFR1*		*PORCN*	*STK36*	*SMPD1*	*CISH*
CDK2	*TEAD1*	*MXI1*	*HEY1*		*MLST8*		*FGFR2*		*RSPO1*	*GLI1*	*MAPK3*	*IL22RA2*
CDK4	*TEAD2*	*MYC*	*HEY2*		*MTOR*		*FGFR3*		*SFRP1*	*GLI2*	*PIK3R1*	*SOCS4*
CDK6	*TEAD3*	*MYCL*	*HEYL*		*NPRL2*		*FGFR4*		*SFRP2*	*GLI3*	*MAPK1*	*CNTF*
RB1	*TEAD4*	*MYCN*	*KAT2B*		*NPRL3*		*FLT3*		*SFRP4*	*GSK3B*	*PIK3CA*	*CNTFR*
E2F1	*PTPN14*		*KDM5A*		*PDK1*		*ALK*		*SFRP5*	*BMP8A*	*PIK3CB*	*CREBBP*
E2F3	*NF2*		*NOTCH1*		*PIK3CA*		*RET*		*SOST*	*IHH*	*MAPK8*	*CSF2*
	WWC1		*NOTCH2*		*PIK3CB*		*ROS1*		*TCF7L1*	*LRP2*	*MAP2K4*	*CSF2RA*
	TAOK1		*NOTCH3*		*PIK3R1*		*KIT*		*TLE1*	*DHH*	*TNFSF10*	*CSF2RB*
	TAOK2		*NOTCH4*		*PIK3R2*		*IGF1R*		*TLE2*	*WNT16*	*DAP3*	*CSF3*
	TAOK3		*NOV*		*PIK3R3*		*NTRK1*		*TLE3*	*SUFU*	*TRAF2*	*CSF3R*
	CRB1		*NRARP*		*PPP2R1A*		*NTRK2*		*TLE4*	*RAB23*	*FADD*	*CSH1*
	CRB2		*PSEN2*		*PTEN*		*NTRK3*		*WIF1*	*CSNK1G1*	*MAP3K1*	*CTF1*
	CRB3		*LFNG*		*RHEB*		*SOS1*		*ZNRF3*	*WNT4*	*RIPK1*	*IL23R*
	LLGL1		*ITCH*		*RICTOR*		*GRB2*		*CTNNB1*	*PRKACA*	*CASP8*	*SPRED1*
	LLGL2		*NCSTN*		*RPTOR*		*PTPN11*		*DVL1*	*PRKACB*	*TRADD*	*IFNLR1*
	HMCN1		*SPEN*		*RPS6*		*KRAS*		*DVL2*	*PRKACG*	*PIK3R3*	*SPRED2*
	SCRIB		*JAG1*		*RPS6KB1*		*HRAS*		*DVL3*	*PRKX*	*CASP10*	*EP300*
	HIPK2		*APH1A*		*STK11*		*NRAS*		*FRAT1*	*PTCH1*	*TNFRSF10D*	*EPO*
	FAT1		*FBXW7*		*TSC1*		*RIT1*		*FRAT2*	*HHIP*	*IKBKG*	*EPOR*
	FAT2		*FHL1*		*TSC2*		*ARAF*		*FZD1*	*SHH*		*AKT1*
	FAT3		*THBS2*				*BRAF*		*FZD10*	*BMP2*		*AKT2*
	FAT4		*HDAC2*				*RAF1*		*FZD2*	*BMP4*		*CLCF1*
	DCHS1		*MFAP2*				*RAC1*		*FZD3*	*BMP5*		*PIK3R5*
	DCHS2		*CUL1*				*MAP2K1*		*FZD4*	*BMP6*		*CBLC*
	CSNK1E		*RFNG*				*MAP2K2*		*FZD5*	*BMP7*		*GH1*
	CSNK1D		*NCOR1*				*MAPK1*		*FZD6*	*BMP8B*		*GH2*
	AJUBA		*NCOR2*				*NF1*		*FZD7*	*SMO*		*GHR*
	LIMD1		*MFAP5*				*RASA1*		*FZD8*	*WNT1*		*IFNL2*
	WTIP		*HDAC1*				*CBL*		*FZD9*	*WNT2*		*IFNL3*
			NUMB				*ERRFI1*		*WNT1*	*WNT3*		*IFNL1*
			JAG2				*CBLB*		*WNT10A*	*WNT5A*		*GRB2*
			MAML3				*CBLC*		*WNT10B*	*WNT6*		*IL19*
			MFNG				*INSR*		*WNT11*	*WNT7A*		*SOCS7*
			CIR1				*INSRR*		*WNT16*	*WNT7B*		*IFNE*
			CNTN1				*IRS1*		*WNT2*	*WNT8A*		*IFNA1*
			MAML1				*SOS2*		*WNT3A*	*WNT8B*		*IFNA2*
			MAML2				*SHC1*		*WNT4*	*WNT10B*		*IFNA4*
			NUMBL				*SHC2*		*WNT5A*	*WNT11*		*IFNA5*
			PSEN1				*SHC3*		*WNT5B*	*WNT2B*		*IFNA6*
			PSENEN				*SHC4*		*WNT6*	*WNT9A*		*IFNA7*
			RBPJ				*RASGRP1*		*WNT7A*	*WNT9B*		*IFNA8*
			RBPJL				*RASGRP2*		*WNT7B*	*ZIC2*		*IFNA10*
			RBX1				*RASGRP3*		*WNT8A*	*WNT10A*		*IFNA13*
			SAP30				*RASGRP4*		*WNT8B*	*WNT5B*		*IFNA14*
			SKP1				*RAPGEF1*		*WNT9A*	*PTCH2*		*IFNA16*
			SNW1				*RAPGEF2*		*WNT9B*	*BTRC*		*IFNA17*
			CTBP1				*RASGRF1*		*AMER1*	*WNT3A*		*IFNA21*
			CTBP2				*RASGRF2*		*APC*			*IFNAR1*
			ADAM10				*FNTA*		*AXIN1*			*IFNAR2*
			APH1B				*FNTB*		*AXIN2*			*IFNB1*
			ADAM17				*RCE1*		*DKK1*			*IFNG*
			DLK1				*ICMT*		*DKK2*			*IFNGR1*
			DLL1				*MRAS*		*DKK3*			*IFNGR2*
			DLL3				*PLXNB1*		*DKK4*			*IFNW1*
			DLL4				*MAPK3*		*GSK3B*			*IL2*
			DNER				*ARHGAP35*		*RNF43*			*IL2RA*
			DTX1				*RASA2*		*TCF7*			*IL2RB*
			DTX2				*RASA3*		*TCF7L2*			*IL2RG*
			DTX3				*RASAL1*		*CHD4*			*IL3*
			DTX3L				*RASAL2*					*IL3RA*
			DTX4				*RASAL3*					*IL4*
			EGFL7				*SPRED1*					*IL4R*
							SPRED2					*IL5*
							SPRED3					*IL5RA*
							DAB2IP					*IL6*
							SHOC2					*IL6R*
							PPP1CA					*IL6ST*
							SCRIB					*IL7*
							PIN1					*IL7R*
							KSR1					*IL9*
							KSR2					*IL9R*
							PEBP1					*IL10*
							ERF					*IL10RA*
							PEA15					*IL10RB*
							JAK2					*IL11*
							IRS2					*IL11RA*
												IL12A
												IL12B
												IL12RB1
												IL12RB2
												IL13
												IL13RA1
												IL13RA2
												IL15
												IL15RA
												JAK1
												JAK2
												JAK3
												LEP
												LEPR
												LIF
												LIFR
												MPL

Footnotes:
ICB = immune checkpoint blockade (1 - four gene panel, 2 - gene panel extended by genes
from seven pathway included in the CIRCLE = the Cancer Immunotherapy Response CLassifiEr) according to:
Gajic et al. Nat Commun. 2022 (doi: 10.1038/s41467-022-31055-3)

Clinically actionable gene panels, N=5

**HRD (Norquist et al. 2018, [27])**	**ICB1 (Gajic et al. 2022)***	**ICB2 (Gajic et al. 2022)***
ATM	*KRAS*	*KRAS*
ATR	*BRAF*	*BRAF*
BARD1	*TP53*	*TP53*
BLM	*BCLAF1*	*BCLAF1*
BRCA1		*PSMB5*
BRCA2		*PSMA6*
BRIP1		*PSMC2*
CHEK2		*PSMD7*
MRE11A		*PSMA5*
NBN		*UBA52*
PALB2		*PSMB2*
RAD51C		*PSMA7*
RAD51D		*PSMB4*
RBBP8		*PMAIP1*
SLX4		*E2F1*
XRCC2		*CALR*
		APOE
		COLEC12
		ICAM1
		ITGA2B
		FGG
		ITGB1
		COL5A1
		COL18A1
		VTN
		GNB3
		CSNK2B
		TUBA1C
		GNAT2
		CCNE1
		NOP56
		TUBB2B
		ZFHX3
		HIST1H2BO
		HIST1H2BK
		H2AFV
		AKT3
		CRKL
		PMAIP1
		CASP2
		FGF4
		STAT1
		CD58
		TEK
